# Experimental study on the impact of indoor air quality on creativity by Serious Brick Play method

**DOI:** 10.1038/s41598-023-42355-z

**Published:** 2023-09-19

**Authors:** Shmitha Arikrishnan, Adam Charles Roberts, Wee Siang Lau, Man Pun Wan, Bing Feng Ng

**Affiliations:** 1 Interdisciplinary Graduate School, Energy Research Institute @ NTU, Nanyang Technological University, 1 Cleantech Loop, Singapore, 637141 Singapore; 2 Future Resilient Systems, Singapore-ETH Centre, ETH Zurich, 1 College Ave E, CREATE Tower, Singapore, 049374 Singapore; 3 School of Mechanical & Aerospace Engineering, Nanyang Technological University, 50 Nanyang Avenue, Singapore, 639798 Singapore

**Keywords:** Mechanical engineering, Cognitive neuroscience

## Abstract

Companies are increasingly asking their employees to find creative solutions to their problems. However, the office environment may reduce an employee’s creative potential. In this study, the role of indoor air quality parameters (PM_2.5_, TVOC, and CO_2_) in maintaining a creative environment (involving lateral thinking ability) was evaluated by Serious Brick Play (SBP), an adaptation of the LEGO Serious Play (LSP) framework. This study was conducted in a simulated office space with 92 participants over a period of 6 weeks. The SBP required participants to address a challenge by building using Lego bricks, and then describe the solution within a given timeframe. The creations and descriptions were then graded in terms of originality, fluency, and build. The results indicated that higher TVOC levels were significantly associated with lower-rated creative solutions. A 71.9% reduction in TVOC (from 1000 ppb), improves an individual’s full creative potential by 11.5%. Thus, maintaining a low TVOC level will critically enhance creativity in offices.

## Introduction

Creativity is involved in everyday thinking through one’s ability to restructure and use knowledge in an unconventional way, such as gardening in the backyard or conducting a complicated task. A common framework for categorising parameters that affect people’s creativity is the Four P’s model (Person, Process, Press, and Product)^1^. ‘Person’ is the individual who is creating, as some people can be more creative than others^2^. ‘Process’ is the procedure used by a Person to develop the Product, and different Processes (such as differing assessment methods) can lead to different outcomes^3^. ‘Product’ is built by the Person and is a result of the Process. ‘Press’ refers to the environment where a Person builds their Product, and this Press refers to conditions that may facilitate or impede the creative process^4^.

Bronfenbrenner’s ecological systems theory characterises different levels of the environment. The theory defines that humans develop through the processes of continuously more complex interactions between individuals and the people, objects, and symbols in their immediate external environment^5^. Reference^6^ investigated the role of physical environment interactions in an individual’s creative potential. The study concluded that a natural environment was rated as relatively higher in creative potential than an artificial environment and ratings of a space’s creative potential predicted creative performance of an independent sample of participants in that space. However, the study mentioned that non-photographable properties such as noise, odour levels, ventilation, and temperature were not taken into consideration in the analysis. Previous research on how the environment could affect creativity focused on the physical attributes, e.g., how individuals use objects present in the environment to solve a problem^7^, rather than the quality of the environment. Indoor environment quality (IEQ) refers to the building’s performance in terms of quality of acoustics, thermal and visual comfort, and indoor air quality (IAQ). While some studies have assessed the influence of noise^8,9^, temperature^10^, and lighting^11^ on creativity, the effect of IAQ on creativity is still largely unknown.

IAQ parameters include levels of gases (e.g., carbon monoxide, carbon dioxide, volatile organic compounds, or formaldehyde), airborne particulate matter (e.g., PM_10_, PM_2.5_), and airborne microbial contaminants (e.g., fungi, bacteria) together with environmental condition parameters (e.g., air temperature, relative humidity, and air movement). Excessive exposure to these air pollutants or abnormal environmental conditions can cause adverse health and psychological effects such as low productivity, sick-building syndrome, and impaired cognitive abilities^12–15^. However, little is known about the effects on an individual’s creative potential. As creativity is a complex ability that involves multiple cognitive processes, it leads to the hypothesis that there could also be an association between IAQ and creativity.

As such, this study seeks to understand how improving IAQ through reduction in TVOC, PM_2.5_ and increase in ventilation rate (changing the “Press” in the 4 P’s model) could affect creativity. To ensure that there are no confounding effects of other environmental parameters, a controlled environment chamber simulating a typical office environment is adopted. In the chamber, IAQ was systematically manipulated for levels of TVOC and PM_2.5_ (through the use of filters) and ventilation rate (by changing the fresh air intake). In addition, to ensure there are no confounding effects of inter-individual differences (the “Person” in the 4 P’s model), the study was a within-participants design, so that all participants experienced all levels of IAQ. Finally, to ensure the creativity task is repeatable and quantifiable (“Process” and “Product” in the 4 P’s model), a new methodology, Serious Brick Play (SBP), is adopted from the Lego Serious Play framework. In the following sections, the development of creativity assessments is discussed in “Development of creativity assessments” section, and the experiment methods used in this study are described in “Methods” section including the chamber, experimental conditions, study population, activity and the statistical analysis approach. In “Results” section, the results are presented followed by discussion and conclusions. This study aims to shed light on the importance of maintaining good IAQ in enhancing the full creative potential of occupants.

## Development of creativity assessments

### Existing creativity tests

According to Ref.^16^, everyday thinking comprises of two creative processes, divergent thinking and convergent thinking. Divergent thinking allows idea generation, where the selection criteria are vague with many possible solutions^17^. In contrast, convergent thinking uses persistence and focus on a well-defined problem to find a single solution^18^. Convergent and divergent thinking can be combined (also known as lateral thinking, which is less discussed and more complex) to allow a set of approaches and techniques to find radically novel approaches to solve problems^19,20^. Lateral thinking ability represents the full creative potential of an individual. However, previous creativity studies use divergent thinking to test creativity with open-ended problems rather than tapping onto lateral thinking ability to test creativity^21,22^.

Reference^16^ employed the Alternative Uses Test (AUT), which requires the participant to think of as many use cases as possible for a simple object as a test of divergent thinking. AUT creativity is measured based on the number of use cases generated and how well the participant performs across sub-categories (e.g., *fluency, originality, flexibility,* and *elaboration*). The test requires participants to have prior knowledge of different use cases which are influenced by different lifestyles and cultural experiences, and therefore has questionable psychometric quality. In particular, the requirement of prior knowledge introduces bias in generating the list of uses^23^. In addition, having a large sample size penalises the uniqueness of each response compared to a small sample size^24^. As an alternative, the Torrance Tests of Creative Thinking (TTCT) is popularly used to measure divergent thinking, subdivided into verbal and figural components^2^. TTCT involves guessing of drawings as part of verbal TTCT and completing a picture as part of figural TTCT. However, the TTCT test fails to measure the utility of ideation creativity, such as the important cognitive process of insight where the moment of comprehension is used to solve problems^23,25^. The AUT and TTCT are based on the concept that creative thinking is stimulated by restructuring and using prior knowledge to generate creative solutions. These conventional tests are designed around divergent thinking but are limited in testing lateral thinking ability. This can be especially problematic, as the outcomes of divergent thinking tests are often overgeneralised as representing all aspects of creativity^23^.

Lateral thinking involves both divergent and convergent thinking. Reference^26^ suggested the principle of knowledge and playfulness, or the imaginative use of knowledge to trigger divergent thinking as the essential component to simulate creativity. A study that tested participants ability to solve a mathematics problem while remembering a list of words showed that they could remember more if they use hand gestures for explanation^27^. Another study showed that hand gesturing led to better learning^28^. It is suggested that using hands to manipulate and construct is a primal way for the brain to use and construct its knowledge of the world^29^. Thus, using hands to gesture and aid in a playful way could potentially unleash creative thinking. Considering the possible link between using hands and creativity, Ref.^30^ described the LEGO SERIOUS PLAY (LSP) method to unleash creativity through a hands-on building process.

### LEGO® SERIOUS PLAY® (LSP)

LSP involves a group of people expressing their thoughts and ideas by building three-dimensional models individually with the LEGO bricks provided. Subsequently, they described the significance of their build to other members of the group. LSP is famous for team building, working out the best solution to a shared challenge, strategy development, and unleashing creative thinking. LSP is an open-source methodology that is made available by LEGO® Group under a Creative Commons licence. It is used as a facilitation methodology involving imagination, discoveries, and design opportunities to address enterprise, team, or personal development problems and is designed to unlock new knowledge and break habitual thinking^30^, providing a new way to trigger lateral thinking and assess creativity.

The method involves four steps: *challenge*, *construction*, *sharing*, and *reflection*. For instance, in a typical LSP session, the facilitator introduces the challenge through related questions. In the construction step, members of the group would individually build a model using LEGO bricks for a specific duration. In the sharing step, members in the group describe their build’s significance on how it addresses the challenge (step 1) with other members in the association. In the final step, reflection, the facilitator and other members in the group would reflect on the shared model (step 2) by asking questions, sharing insights, and identifying patterns together with the builder. LSP as a method of eliciting creativity has the advantages of requiring illustrative explanations and allowing a range of solutions to a challenge. However, while LSP has been used extensively for teambuilding group exercises, the method does not have a quantitative assessment component and cannot systematically assess creativity.

There are numerous methods available to quantify creativity. However, the quantification method used should adapt and be feasible to the individual unique construct of the creativity test^3,31–34^. Based on the Four P’s model of creativity^1^, an assessment of a ‘‘product’’ (such as the built outcome of LSP) can be performed using three grading systems, namely, Creative Solution Diagnosis Scale (CSDS)^35^, Consensual Assessment Technique (CAT)^36^ and Creative Product Analysis Matrix (CPAM)^37^, giving a quantifiable score for the output of the LSP test for creativity. Among the three, CPAM is a cost-effective method with an easy-to-follow guideline for non-expert judges without compromising the structure of the quantification process (see “Statistical procedures” section).

## Methods

This study was approved by the Institutional Review Board of Nanyang Technological University, IRB-2017-06-014, and all methods were performed in accordance with the relevant guidelines and regulations per the Declaration of Helsinki. Written informed consent was obtained from all subjects to take part in the study.

A single-blind experiment was adopted for this study. It included repeated measurements of creativity on the same participant while switching the environmental chamber between six pre-defined sets of IAQ conditions to which each participant was exposed. The participants ran through an experimental round of 7 weeks, spending 1 day per week in the chamber. The first week (week 0) was to train the participants to be familiar with the experimental protocol, whereas the following 6 weeks were examinable weeks during which the participants were exposed to one set of IAQ conditions per week and examined for creativity.

### Environmental chamber

**Figure 1:**
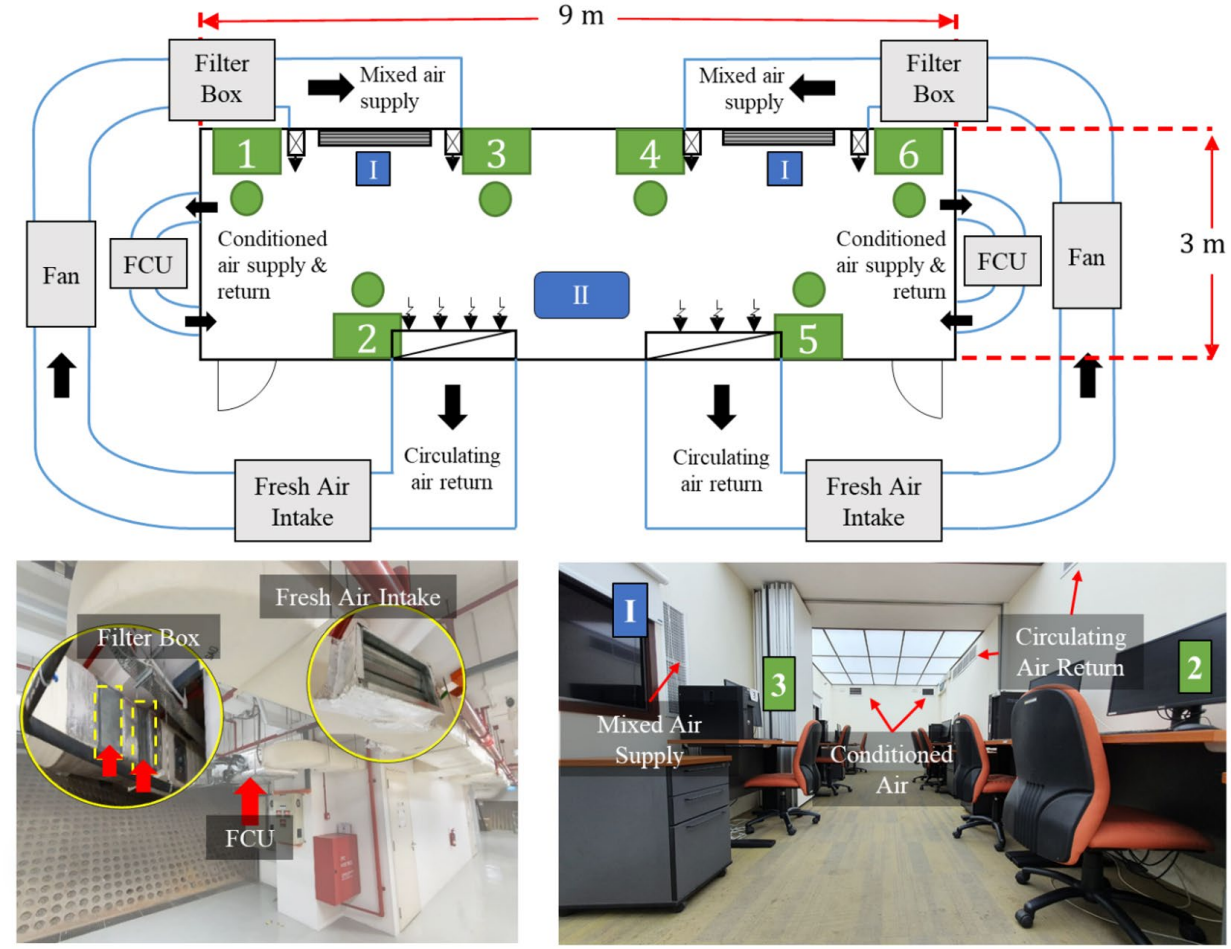
Schematics of the environmental chamber. Participants were allowed to choose any of the numbered desk (green) on the first day and that was assigned to them for 6 weeks. The illusion of windows is indicated as (I). The IAQ indicators were measured on a cart at a raised height of 1.5 m from the ground in the middle of the room located at (II), 1 m away from the participants. The fan runs at 300 L/s and FCU indicate fan coil unit operates at 150 L/s. The filter box was designed with hidden slots dedicated to Carbon and PM filters and the adjustable fresh air intake allows for variation in the ventilation rate.

The environmental chamber used in this study is shown in Fig. 1 with internal dimensions of 9 m (L) × 3 m (W) × 2.4 m (H) and located in the basement of a laboratory building at Nanyang Technological University, Singapore. The chamber’s side walls are constructed with 12 mm-thick calcium silicate boards with rock wool insulation sandwiched in-between. The surfaces of the wall were painted in white colour (emulsion paint) and the concrete flooring was covered with nylon carpet tiles. The ceiling was fully covered by dimmable LED lighting fixtures. One of the longer sides of the chamber had two flat-screen LCD TVs covered with roller window blinds with a spring chain system to mimic windows to reduce the feeling of confinement^38,39^. The internal space of the chamber was furnished to resemble a typical office environment with six working desks. Each desk was furnished with a desktop computer and a set of LEGO bricks for the creativity test.

The configuration of the chamber is symmetrical in length. The chamber utilised two individual air-conditioning and mechanical ventilation (ACMV) systems as shown in Fig. 1. On each half of the chamber, conditioned air was provided by a fan coil unit (FCU) having a cooling capacity of 1.5 kW and a constant volumetric flow rate of 150 L/s. The conditioned air passes through an air diffuser into the chamber and is returned through a return air grill on the shorter side wall. The air in the chamber was circulated by a circulating air loop driven by a fan with a volumetric capacity of 1000 L/s controlled by a variable speed drive. Each of the circulating air loops had a fresh air intake with an adjustable dampener to allow for ventilation rate adjustment and a filter box with three filter slots to allow for a maximum of two PM filters and a carbon (TVOC) filter to be fitted in. By fitting different combinations of filters into the filter box, PM and TVOC levels in the chamber could be varied. The fan speed was varied to compensate for the different pressure drops due to the insertion of filters such that the circulation rate remained constant at 300 L/s for all tests.

During the experiment, the air temperature in the environmental chamber was maintained at 24–25 °C with a relative humidity of 50–60%. The light intensity at the desktop level was maintained at 500 lx, in line with^40^ (300–500 lx), and the noise level was maintained at 54.8 dBA (8-h average) in line with^41^ (90 dBA, 8-h day exposure). The floor carpet was vacuum cleaned before the start of each round (without using any cleaning agent).

### Experimental conditions

The experimental conditions of the 7 weeks for each round are summarised in Table 1. Week 0 was the training week during which no filter was inserted into the filter box. In the following 6 weeks, IAQ conditions were varied by 3 methods: The baseline condition consisted of a low ventilation rate of 0.5 air changes per hour (ACH), or 9 L/s, (LV in Table 1). This condition was repeated twice in each round so that any learning effect (participant increasing in proficiency in the task over time) could be detected and be offset from the effects of changing the IAQ conditions in subsequent data analysis. An increased ventilation rate of 1 air change per hour (ACH) or 18 L/s, denoted as ‘HV’ in Table 1 was used in the other conditions. This ventilation rate corresponds to the minimum fresh air supply rate of 0.6 L/s-m^2^ for offices recommended by the Ref.^42^. Pollutants were reduced by inserting PM filters (denoted as ‘PM’ in Table 1) and inserting carbon filters (denoted as ‘C’ in Table 1) or inserting both filters (denoted as ‘PM-C’ in Table 1). The PM filter used was a combination of G4 (CAMFIL 30/30 Panel Filters) and F8 (CAMFIL EcoPleat G 3GPPS-12244-F8) particulate filter, which has a rating of ePM1 at 70%^43,44^. The carbon filter used was a CAMFIL Gigapleat NXPC C3^45,46^ for TVOC reduction. The ventilation rate was measured by the tracer gas decay method using CO_2_ as the tracer gas.

**Table 1:**
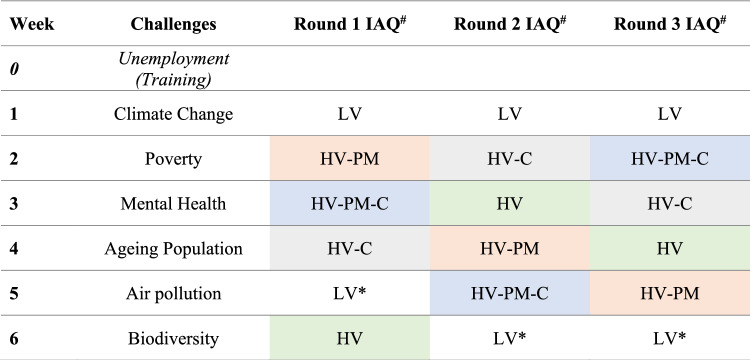
The sequence of given challenges and varying IAQ condition settings.

^#^IAQ condition setting: *LV* low ventilation baseline (0.5 ACH), *HV* high ventilation (1 ACH), *PM* PM filter inserted, *C* carbon filter inserted.

*Repeated condition.

### Study population

The study population consisted of 92 university students (undergraduate and postgraduate) of diverse backgrounds. The population comprised an equal number of males and females, aged between 21 and 30 years, from different academic disciplines. Participants were screened for eligibility using a pre-test questionnaire. Eligible participants were healthy individuals without any allergies, chronic diseases, claustrophobia, learning disorders, or neurological disorders. Individuals who were taking psychoactive medication and current or social smokers were also excluded from the study.

The participants were divided into three rounds of study following the same protocol. For each round, participants attended a training session (Week 0) on the first week to get familiarised with the experimental protocol and dampen any potential learning effects^47^. Participants in each round were rotated weekly. The weekly rotation requires participants to report to the chamber on the same day of each week, i.e., Monday participants participate in the study on each Monday of the 7 weeks. Out of the 92 selected participants, 2 dropped out before completing all 7 weeks of experiments. Data points from 3 participants were removed from the dataset due to minor technical issues that occurred during the experiments. Data collected from 87 participants (522 data points) were used in the subsequent data analysis. The results from the training week (Week 0) were not included in the analysis.

### Instrumentation

IAQ parameters were monitored continuously in the chamber during the experiment, using the instruments summarised in Table A-1. Monitored IAQ parameters include airborne bacteria level (cfu/m^3^) and airborne fungi level (cfu/m^3^), carbon dioxide (CO_2_, ppm), carbon monoxide (CO, ppm), formaldehyde (HCHO, µg/m^3^), ozone (O_3_, ppm), airborne particulate matter (PM_2.5_, µg/m^3^), relative humidity (RH, %), the total volatile organic compound (TVOC, ppb) and air temperature (T, °C). The daily 8-h average exposure, according to the Ref.^48^, presented in Table 2 was used for analysis.

**Table 2:** Levels of IAQ parameters in the environmental chamber under different IAQ condition settings.

		LV	HV-C	HV-PM	HV-PM-C	HV	LV*	SS 554 (2016)^1^	WHO (2016)^2^
IAQ parameters									
PM_2.5_ (µg/m^3^)	8 h TWA	15.5	10.0	2.4	4.0	12.2	10.7	37.5	25
	Max	35.5	21.7	2.8	10.1	29.3	16.1		
	Min	14.8	10.6	1.2	1.2	6.3	6.7		
TVOC (ppb)	8 h TWA	668.5	280.8	535.3	342	578.3	565.7	1000	–
	Max	735.3	312.9	719.8	403.3	831.8	669.9		
	Min	533.6	241.4	538.9	298.7	333.8	579.8		
CO_2_ (ppm)	8 h TWA	801.9	700.1	755.5	742.1	695.4	748.1	700 above outdoor	300
	Max	965.0	810.3	913.6	914.9	805.9	963.0		
	Min	768.4	694.7	756.1	741.6	740.1	764.4		
Other parameters									
Formaldehyde (ppb)	8 h TWA	9.0	9.5	9.4	9.6	9.8	8.9	81.0	81.0
Carbon monoxide (ppm)	8 h TWA	2.4	2.3	2.5	2.4	2.4	2.5	9.0	9.0
Bacteria level (cfu/m^3^)	Ave	79.5	105.6	185.7	150.0	184.1	150.5	1000.0	–
Fungi level (cfu/m^3^)	Ave	162.3	56.2	134.8	28.1	129.8	132.0	–	–

All 3 rounds are combined accordingly to the IAQ condition settings. The maximum and minimum values are obtained from the combined data of the 3 rounds. Bacteria and fungi levels are based on the average from the morning and afternoon sampling per study day. Data from PM_2.5_ was calibrated to the dust present in the environmental chamber before the start of the study. Data from TVOC are based on Toluene standard. Outdoor CO_2_ level is approximately 450 ppm. SS 554 limit indicates the reference IAQ levels used in the study and WHO indicates the international reference levels.

^1^Reference^48^.

^2^Reference^49^ and *TWA* time weighted average.

### Serious Brick Play (SBP) design

Each participant was given a bag of an equal amount of LEGO® CLASSIC bricks with colour variations. The compositions of bricks in all bags were similar in terms of numbers for each specific size and specialised pieces (e.g., doors, windows, wheels). The bags of LEGO bricks assigned to the participants were rotated for each week’s study to ensure that participants were exposed to random selections of bricks to reduce brick familiarity.

Due to the shortcomings of the current assessments, we propose the SBP method, built upon the LSP methodology, as a way of quantifiably assessing lateral creative thinking. Each of the four core steps of LSP was modified to fit into a quantitative assessed framework, where the participants would complete three core steps: *read*, *build*, and *describe*. *The fourth core step*, *reflection* was replaced with a *post-test assessment*. The key differences and similarities between the two methods are summarised in Table A-2.

The first step, *challenge*, requires a universal problem that caters to the diverse expertise of the participants while not requiring any specialised knowledge to fulfil the task. Global issues of broad emphasis (e.g., climate change) were identified as a suitable theme. However, it is unlikely for all participants to have the same expertise/experience on the topic. Hence to reduce bias caused by different levels of expertise/experience, participants were required to read a document with a comprehensive background summary on the topic. The background information was sourced from various channels, including the United Nations Foundation, Worldwide Fund for Nature, National Aeronautics and Space Administration, local newspapers, World Health Organisation Public Health, the Mental Health Foundation, and Nature Communications. After reading the challenge carefully, participants were instructed to build their models to express a solution to the challenge (see Supplementary Appendix B).

In the second step of *construction*, participants built the model(s) to express their ideas about the solution to the given challenge. For each of the 6 test weeks, a different challenge was presented (climate change, poverty, mental health, ageing population, air pollution, and biodiversity, see Table 1). Participants were instructed not to search for any information or solution online as they build the model.

In the third step, *sharing*, participants described their model(s) by explaining the choice of colour (if any) and how it was related to the given challenge. Since the nature of SBP was to test creativity within individuals rather than a collective group effort, individual participants were asked to document the description in writing instead of presenting it to a group of people. This step allows within-individual convergent thinking, choosing and describing the way that their solution is the best way to answer the challenge. Steps 1 to 3 took 40 min to complete (compared to LSP, where a minimum of two hours would be needed). This marked the end of participants’ involvement in the SBP design. Besides the written description, photographs of the built models were taken from different views (front, side, back, and isometric view) by the experiment moderator. The photographs were attached to the written descriptions for grading in the next step. The final step, *reflection* is a post-test assessment. The post-test assessment involved a panel of randomly picked graders to score the models built and the descriptions given by the participants.

### Statistical procedures

#### Selection of grading model

Creative product analysis matrix (CPAM) is a three-factor model (Novelty, Resolution, and Elaboration & Synthesis) of creativity^50^. The 3 factors are expanded to nine facets (Originality, Surprise, Logical, Useful, Valuable, Understandable, Organic, Well-crafted, and Elegant) used to grade creativity. The method was validated by Ref.^37^ showing strong construct validity when assisted untrained judges make informed judgments following CPAM. However, the CPAM is a framework, and not a scale. Previous scales have been proposed, for example CPSS has 55 questions^51^. The revised version in Besemer and O’Quin^37^ has 43 items. The disadvantage of this scale is that it is very time consuming for the judges to rate each product. As our study involved a large number of participants completing six trials each, we endeavoured to formulate a short-form scale that would not have the pitfalls of the AUT, TTCT, or the CPSS.

#### Grading guidelines formulation and grading panel reliability

**Figure 2:**
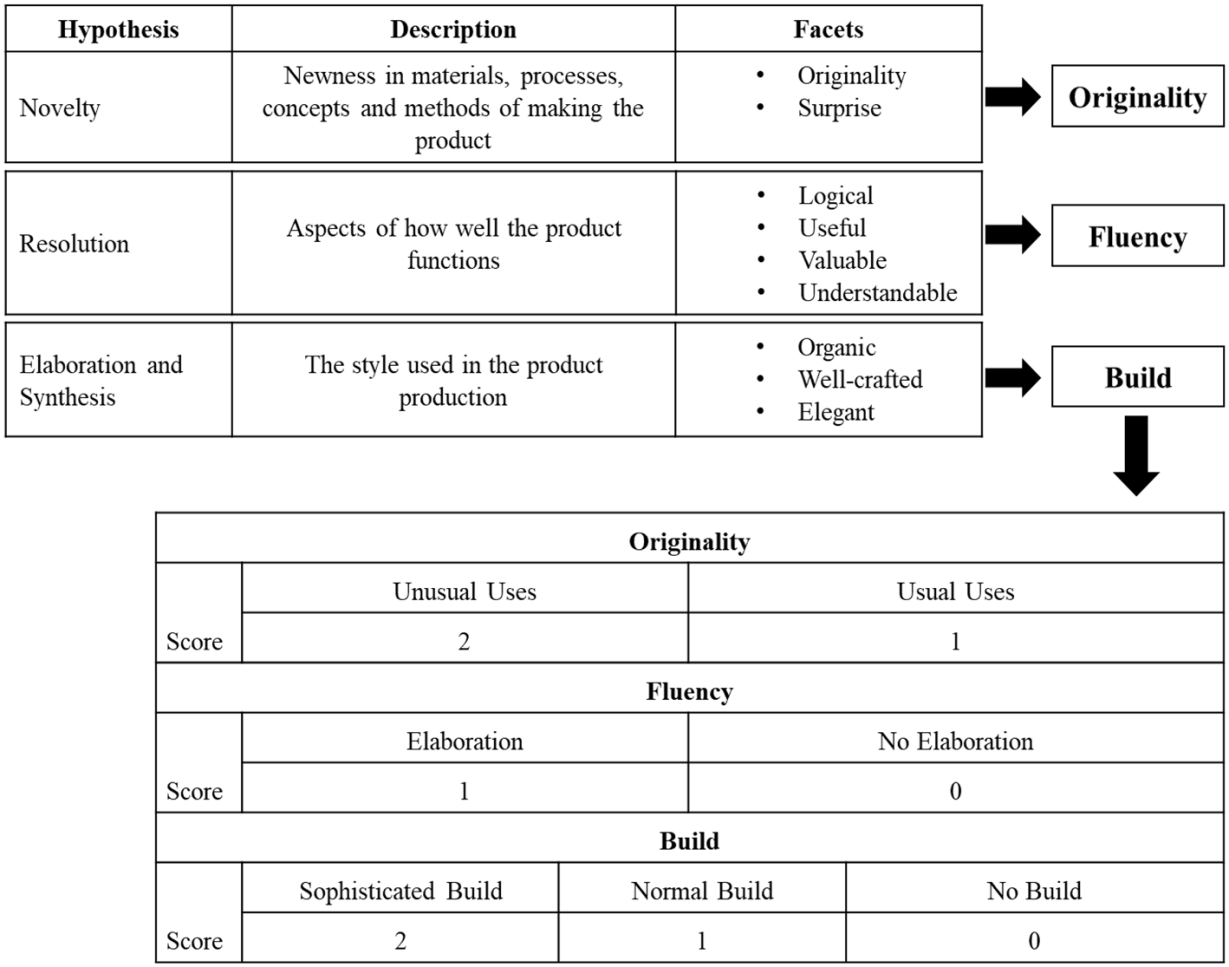
Details on how CPAM was adapted to Serious Brick Play (SBP) methodology to establish a set of grading guidelines. Each of the criterion’s facets of CPAM is summarised into a factor applicable to SBP that is further broken down into gradable indicators. Supplementary Appendix D indicates what the grading panel considers usual and unusual uses.

The grading guidelines were formulated based on the CPAM guidelines but catered more toward SBP’s methodology. Each factor (novelty, resolution and elaboration and synthesis) in CPAM was translated into a term related to SBP. For example, ‘Novelty’ from CPAM was translated to ‘Originality’ in the grading guidelines used in this study (see Fig. 2). Originality was divided into *usual* solutions (scoring one point) or *unusual* solutions (scoring two points). Disagreement about what was considered usual and unusual led the panellists to develop a comprehensive list of usual solutions/ideas for each challenge (see Supplementary Appendix C). A solution was considered unusual when the item could not be categorised in the list. ‘Fluency’ was divided into *elaboration* (scoring one point) and *no elaboration* (scoring no points). If participants had fulfilled all sub-points under the specified indicator, they would receive a score for elaboration (see Supplementary Appendix C). As the most important part of the Lego process, ‘Build’ was split into three levels: *sophisticated*, *normal* and *no build* (i.e., a low effort build). There was no strict definition for the three levels. Instead, it was left to the graders’ discretion since the activity aimed to quantify the participants for their creativity. However, in a dilemma, the graders could refer to the brief guide provided for reference (see Supplementary Appendix D). With this set of grading guidelines (Fig. 2), graders could independently grade each item with a minimum of 1 and a maximum of 5 points.

**Figure 3:**
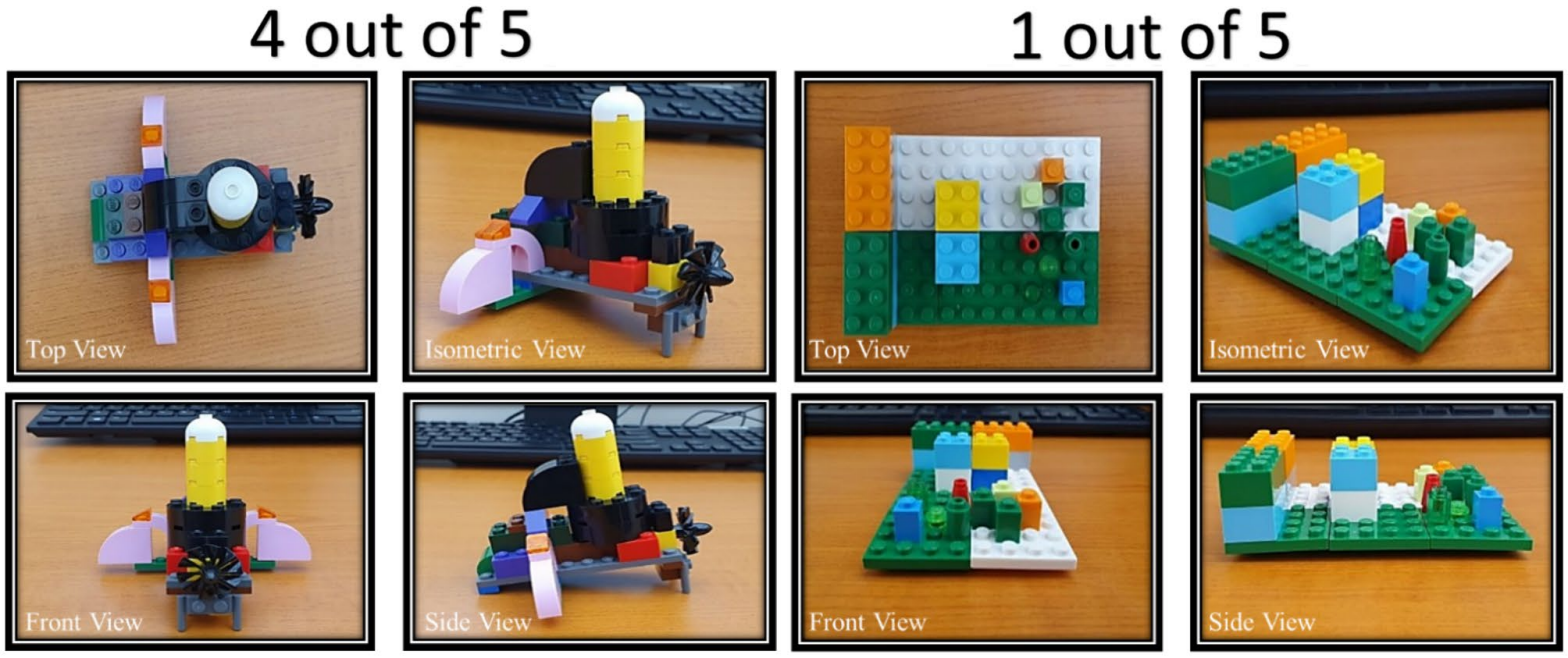
Example builds scored the highest and lowest points in accordance with the SBP guidelines. The builds were randomly selected. The score represents the rounded average across seven graders. See Supplementary Appendix E for the participant description of the models.

The grading panel was made up of 7 randomly selected adults who had no prior knowledge of SBP. Structured training was given to the panel to familiarise themselves with SBP and the grading guidelines. The grading panel was kept blind to the overall experimental design and participant protocols to prevent biased scoring. The best-scored build and the worst-scored build averaged across the grading panel, are shown in Fig. 3. The robustness of the grading guidelines was tested with 20 trial samples (from week 0) from the same population for the panel to score. The validity of the grading guideline was determined by testing the inter-rater reliability using Intra-Class Correlation (ICC). ICC is an index that reflects the degree of correlation and agreement between measurements^52^. The assessment was done with the total scores obtained from each grader. ICC is determined using Eq. (1) (Hallgren 2012^53^).

1 $$\begin{array}{c}{X}_{ij}=\mu +{r}_{i}+{e}_{ij},\end{array}$$

where *X*_ij_ is the rating provided to participant i by grader j, *µ* represents the mean of the true score for variable *X*, r_i_ is the deviation of the reuse score from the mean for participant i and e_ij_ is the measurement of error.

The ICC model used is a two-way model with type as consistency. The interpretation of the ICC estimate is based on the lower bound, 95% confidence interval; values of < 0.50, > 0.50 and < 0.75, > 0.75 and < 0.90 or > 0.90 were classified as poor, moderate, good, and excellent reliability, respectively^52^. This indicates the degree of agreement among the graders.

#### Analysis

Although the IAQ in the chamber was controlled using filters and manipulation of fresh air ventilation rate, pollutant levels can fluctuate based on the occupants of the room and outdoor air quality. To account for this, the analysis of IAQ effects on creativity score was performed in two parts. We first tested the effects of IAQ condition (HV, HV-C, HV-PM, HV-PM-C, LV, LV*) on the average creativity score using a linear mixed effects model. The linear mixed effect model uses restricted maximum likelihood and a non-linear optimiser to predict the effects. Comparisons between conditions were performed using post-hoc contrasts with Dunnett’s correction for multiple comparisons. p-values < 0.05 are considered statistically significant.

We then tested the effects of individual IAQ parameters (TVOC, PM_2.5_, and CO_2_) on the average creativity scores with linear mixed regression models. The regression models adopt a hierarchical model structure: base model built on covariates (Model 0: formaldehyde, carbon monoxide, bacteria count, and fungi count, participant ID as the random intercept, and week (scaled and centred) as the random slopes). Models 1, 2, and 3 each were built on IAQ parameters: PM_2.5_ + Model 0, TVOC + Model 0, and CO_2_ + Model 0, respectively. The best-fitted model was determined by using Akaike Information Criterion (AIC). AIC compares similar competing models to determine the maximised model’s log-likelihood. It is considered maximised when a model is more than two AIC units lower than another model^54^. AIC cumulative weight expresses the percentage proportion of the total predictive power to the model compared to all other models^54^. The lowest AIC value and the highest cumulative weight conclude the best fit linear mixed regression model with the highest quality (least amount of information lost by a given model). It is to be noted that models that satisfy the AIC criteria suggest a strong association between creativity score and the IAQ parameter.

## Results

The convergent and discriminant validity of the SBP was tested against the AUT with a subset of participants and showed that the SBP measures a distinct creativity construct (see Supplementary Materials, Tables A-6, A-7, and Supplementary Information A-8). The ICC coefficients were estimated separately for each of the three experimental rounds to assess inter-rater reliability. ICC allows the measurement of the strength of inter-rater agreement for ordinal scales. Thus, a high ICC indicates high similarity between panellists. The psych::ICC() function in R (version 4) was used to compute ICC estimates and their 95% confidence interval (CI) based on a mean rating (k = 7), absolute agreement, and the two-way random-effects model. Based on the lower bounds of 95% CI, as summarised in Tables A-3, the level of reliability of the three rounds of experiments ranges between “moderate” to “good” reliability^52^. Accordingly, we took the mean score across the 7 graders as the final creativity score per participant per condition.

### By-condition analysis

**Figure 4:**
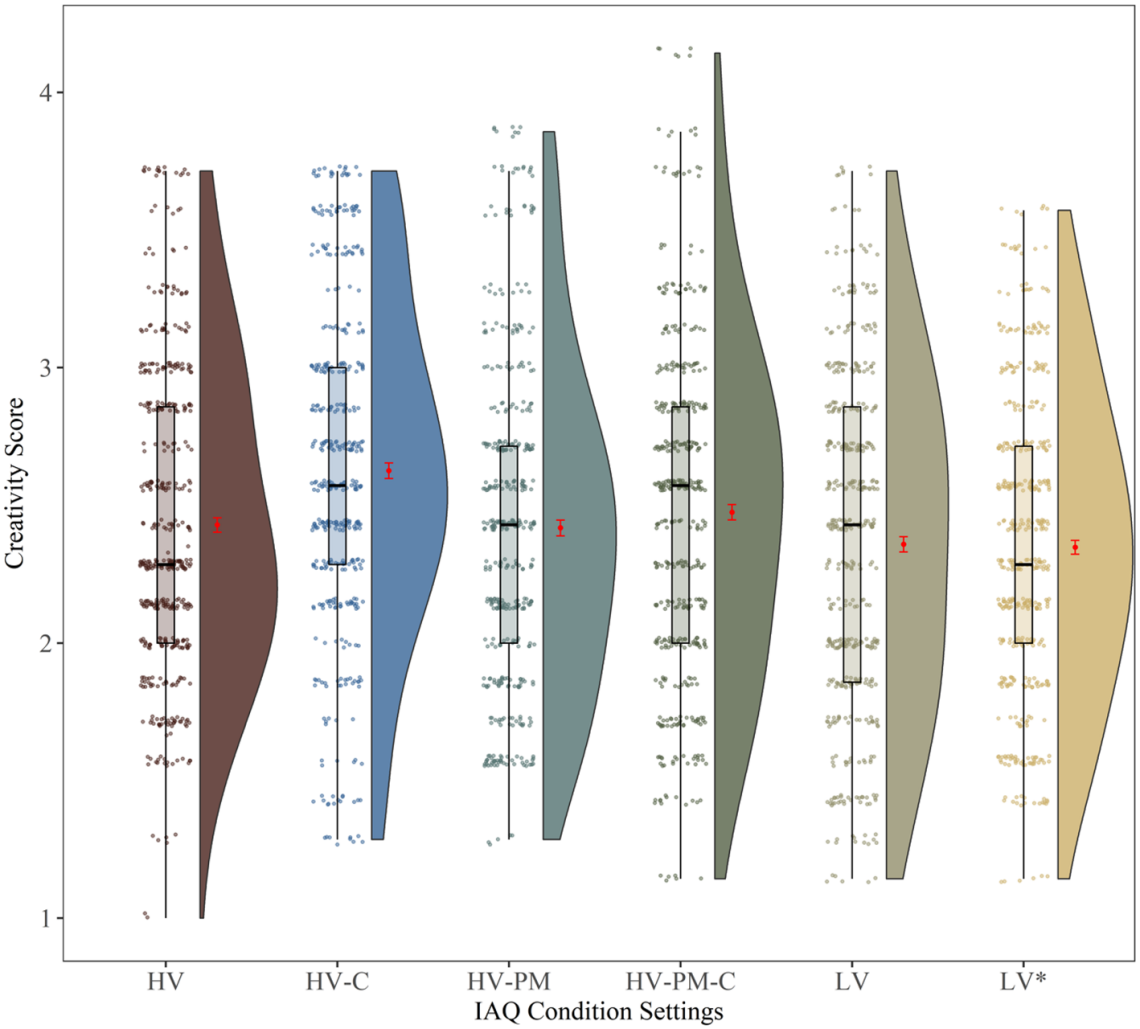
Raincloud plot displaying the linear mixed effects model of creativity score versus IAQ condition settings. The plot consists of a probability density plot, boxplot, data points and the error bar in red. In the boxplot, the line dividing the box represents the median of the data, the ends of the box represent the lower and upper quartiles, and the extreme lines attached to the box represents lowest and highest values among the data points excluding the outliers. The raw data points are the mean scores of 7 raters for each condition. The error bar represents mean (red dot) and standard error of mean (± SEM). Participant ID was treated as a random intercept.

The results of by-condition analysis are shown in Fig. 4. The raincloud plot shows HV-C as the highest mean creativity scores among other conditions tested, although the combined HV-PM-C condition had the widest range and included some of the highest individual creativity scores. The linear mixed model included IAQ *condition* as a fixed factor and *participant ID* as a random intercept. Since the low ventilation baseline condition (LV) was repeated as LV*, they were combined as LV^2^. There was a significant main effect of condition, F(4, 431.22) = 4.18, p = 0.002. Post-hoc contrasts were performed to explore this effect.

The post-hoc contrasts examine the differences between the various conditions with the baseline low ventilation condition (LV^2^), reported in Table 3. The carbon filter (HV-C) condition showed a significantly higher mean creativity score than the LV condition (HV-C β = 0.266, p < 0.001). No other conditions showed significant differences from the LV baseline condition. It is unclear what specific changes in the IAQ cause the HV-C condition to give significantly higher creativity scores than the baseline condition, considering the HV-PM-C condition (which combines both carbon and PM filters) did not show a significant improvement in creativity. When the filters are changed, there are multiple effects, including changes in TVOC, CO_2_, or PM_2.5_. These changes could also be caused by outside environmental changes. Therefore, more complex regression models of our data were tested.

**Table 3:** Least-square means model was used to compare the IAQ condition settings mean to the baseline HV condition setting.

Contrast	Estimate	Standard error	p-value
(HV-C)-LV^2^	0.266	0.07	< 0.001*
(HV-PM)-LV^2^	0.046	0.07	0.847
(HV-PM-C)-LV^2^	0.113	0.07	0.252
HV-LV^2^	0.064	0.07	0.694

The p-value < 0.05* is considered statistically significant after performing the Dunnett correction.

### Hierarchical regression model

The hierarchical linear mixed regression model analysis was performed in the open-source statistical package R (version 4) using the function lme4::lmer(). The residuals are normally distributed and homoscedastic for all linear mixed regression models. Effect sizes based on F tests were calculated from partial eta-squared using Ref.^55^ *f: f* = 0.10 is a small effect, *f* = 0.25 is a medium effect, and *f* = 0.40 is a large effect.

**Figure 5:**
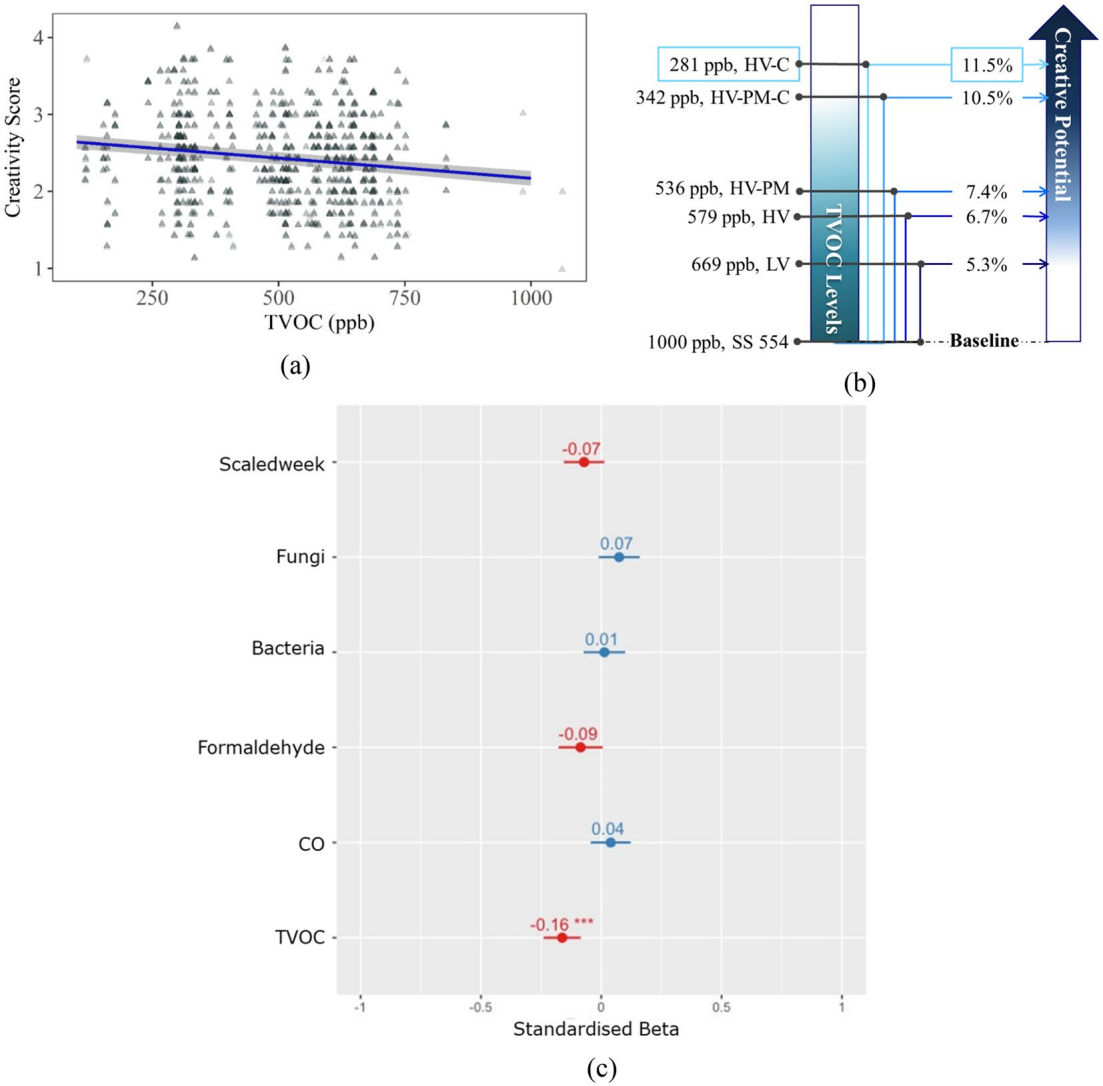
(**a**) Linear mixed regression model estimates of TVOC on creativity scores. The shaded band represents a 95% confidence interval testing levels of TVOC on creativity scores. The triangles represent that data points. (**b**) Impact of different TVOC levels on an individual’s creative potential. Each of the reduced TVOC levels with reference to SS554, Singapore Standard Council^48^ indicates the percentage increase in the creative potential of an individual. For example, a reduction of 719 ppb from SS554 level indicated HV-C IAQ condition setting relates to an increase in 11.5% of creative potential in an individual. (**c**) Forest plot of Standardised Estimates of model 2 indicating TVOC has a significant negative effect on creativity scores.

By comparing the AIC of models 0, 1, 2, and 3, the best-fitted model was determined as Model 2, with an AIC value of 919.7 (the lowest among all models), carrying 100% of the cumulative weight (highest possible AIC weightage), and the only model that was significantly different to the base model 0, χ^2^ = 16.96, p < 0.0001. This was used for further interpretation (refer to Table A-4, Fig. 5c).

Further examination of Model 2 showed a significant negative effect of TVOC on creativity scores with a small effect size (standardised beta =  − 0.16, p < 0.001, *f* = 0.20). A sensitivity analysis was conducted to check the robustness of this finding (Fig. A-5 in the Supplementary Materials), which showed that the effects of TVOC remained when controlling for PM_2.5_ and CO_2_. The linear relationship between creativity and TVOC is depicted in Fig. 5a, where a 10% decrease in TVOC levels is associated with an improved creativity score of 1.6% (obtained from the statistical estimate). Predicted percentage changes in creativity scores were calculated for TVOC values from the maximum of 1000 ppb (reference level of SS554) for each of our conditions (Fig. 5b). These predicted values suggest a potential improvement in creativity scores of approximately 12% if a building’s IAQ is improved from the acceptable maximum TVOC to the levels achieved in our study using commercially available filters.

## Discussion

Creativity is an essential cognitive ability that affects one’s daily lifestyle choices and economic progression. Among the factors that affect creativity, it is also important to note that the environment where creativity is undertaken plays a key role. Previous studies have discussed the optimum physical attributes of an ideal creative environment but have lacked an examination of IAQ and the effects on an individual’s full creative potential^6,56,57^. The data from our study suggests that relatively low concentrations of TVOC in the environment (within international standards) can impact an individual’s creative potential. For example, a 50% decrease in TVOC (from 1000 to 500 ppb) concentration levels due to removing common sources, like perfume, air freshener, and aroma diffusers, would bring about a 7% improvement in creativity as shown in Fig. 5b.

Reference^58^ highlighted the notion of Henri Poincaré (1854–1912), a French mathematician, who went into the forest for creative motivation to find solutions to his mathematical problems as an illustration of the environment influencing creativity. Similarly, the results from this study show that one underlying aspect of an environment that can affect creativity is the chemical concentration of the pollutants in the room. Instead of changing the physical attributes of the environment, improving the air quality could be an economical solution to improve occupants’ creativity. These results build upon the existing work by Allen et al.^12^, where low TVOC and CO2 were reported to affect occupants’ cognitive functions in an enclosed environment.

The study examined undergraduate and postgraduate students only, thus the outcome is skewed towards a young, educated population. Further work could be extended to different age groups, such as the elderly and children. However, the building challenge might need to be modified for these age groups. The key component of SBP is for the participants to understand the content presented in the building challenges. Hence, participants below the age of 15 might have difficulty comprehending the building challenges. If studies were to adopt SBP for participants below the age of 15, simplification of the building challenges is required. It is vital to choose a challenge in line with the participants’ competence (neither too easy nor too difficult). In addition, the IAQ data from this study is limited to concentrations that are found in typical clean office environments and did not achieve concentrations close to standard limits. Hence the delta between the “clean-air” (PM and Carbon filter) conditions and “poor-air” (low ventilation 0.5 ACH) conditions is not large. This could have masked the potential effects of PM_2.5_ and may have limited the effects of TVOC. Variations in outdoor pollutant concentrations may have also had an effect. For example, the mean TVOC level seen in the HV-PM-C condition (where both PM and carbon filters were used) was 22% higher than in the HV-C condition (where only the carbon filter was used). Further work could artificially increase the pollutant levels in the room to better control for this possibility.

Previous studies have utilised tests dedicated to stimulating divergent thinking to understand the mechanisms of creative thinking. However, the main disadvantage of divergent thinking creativity tests is using prior knowledge, which has a biasing effect over diverse cultures and experiences across different individuals. This bias can hinder the accuracy of assessing the divergent thinking ability of an individual. Therefore, this study established a new method of assessing creativity through the implementation of SBP through three core steps (Read, Build, and Describe). This method attempts to remove bias observed in previous creativity tests by providing participants with the same background information and insights into the building challenge from a global to a local perspective. Hence, SBP coupled with a robust grading system allows for the assessment of creativity in a reproducible way.

Part of the SBP methodology involves rotating the set of bricks across participants for each building challenge, such that participants would receive a different set of bricks each week. Each set of bricks contained similar items to the other sets but could vary in colour, size, and shape. This was to minimise the chance of repeatability of structures and familiarity with the brick set. However, the different variations of bricks between the sets could affect participants’ ideas on the challenges. Therefore, to improve the SBP protocol, each participant could be assigned to a unique building challenge (instead of the same building challenge across all participants weekly) along with the set of bricks that would be rotated over the weeks instead. Alternatively, a more extensive standardised set of bricks could be used throughout the different conditions. For example, the LSP methodology has a recommended starter set of 214 bricks and numerous standardised add-on sets to allow matching across participants.

The qualitative output (built photos and description) of SBP was graded with seven random graders, with a guideline modified from CPAM to suit SBP’s core steps. The modification of CPAM was required because creativity is not a unitary measure, and the quantification should match the methodology and theoretical aims of research that are unique to the study^59^. The guideline went through a few iterations to ensure its robustness to cater to different building challenges. The generalisability of the results is limited somewhat by the repeatability of the building challenge provided. The building challenges were based on popular global issues to establish neutrality for participants from different backgrounds and experiences. Hence the building challenge could be used across different groups of people. However, the repeatability of the building challenge to the same group of people is highly discouraged due to familiarity with the challenges. This means that test–retest reliability statistics could not be computed. While our scoring showed moderate to good reliability across scorers, future studies should attempt to replicate this technique to allow measurement of other reliability and validity metrics.

## Conclusions

Creativity, a complex set of idea-generating or imagining behaviours encompassing numerous sub-processes of the brain, is typically tested by examining an individual’s divergent thinking ability. By modifying the LSP paradigm, SBP examines a more general measure of creative ability with hands-on learning through LEGO bricks. The physical outcome (build) and written description can then be graded to quantify participants’ creative ability. Within this study, the SBP method was used to test the impact of IAQ on creativity, where reducing the TVOC concentration showed an increase in creativity score. However, reducing PM2.5 and increasing ventilation rate did not show robust effects.

Since this paper is the first of its kind correlating IAQ pollutants with creativity, there is more scope for future research. The method used to study creativity among participants was interactive and hands-on; it did not allow for individual cognitive processes to be studied. Further study is needed to determine the specific mechanism linking pollutants and creativity. The work of creative cognition is still a new field and using non-invasive aids such as electroencephalography to study the brain responses while participants are engaged in SBP could help researchers understand how hands-on, playful tests stimulate creativity.

### Supplementary Information

Supplementary Information.

## Data Availability

The authors confirm that the data supporting the findings of this study are available within the article.
