# Supplementary material for: Experimental study on the impact of indoor air quality on creativity by Serious Brick Play method
Source: Sci Rep. 2023 Sep 19;13:15488. doi: 10.1038/s41598-023-42355-z (PMC10509215; doi:10.1038/s41598-023-42355-z)
Supplement: Supplementary file 1 — Supplementary Information. [file 41598_2023_42355_MOESM1_ESM.docx]

Appendix A: Supplementary Tables and Figures

**Table A-1**: List of equipment used to measure the indoor air pollutants

| Equipment | Operating Range | | Indoor Air Pollutants |
| --- | --- | --- | --- |
| Graywolf IQ-610 | CO_2_ | 0 – 10,000ppm  Accuracy: +/-3%rdg +/-50ppm | CO_2,_ CO, O_3,_ Temperature and Relative Humidity |
|  | CO | 0 – 500ppm  Accuracy: +/-2ppm<50ppm, +/-3%rdg >50ppm |  |
|  | O_3_ | 0 – 1ppm  Limit of detection: 0.02ppm |  |
|  | Temperature | -10℃ – 70℃  Accuracy: +/-0.3℃ |  |
|  | Relative Humidity | 0 – 100 %rh  Accuracy: +/-2%rh <80%rh (+/-3%rh > 80%rh) |  |
| NanoScan SMPS Nanoparticle Sizer-TSI 3910 | 10 - 420 nm  Accuracy: +/-10% at 0.25lpm | | PM_2.5_ |
| Optical Particle Sizer (OPS)-TSI 3330 | 0.3 - 10 µm  Accuracy: +/-5% at 0.5µm | |  |
| Shinyei Multimode Monitor-FMM-MD | 10ppb – 1000 ppb  Accuracy: +/- 4 ppb < 40ppb,  +/-10% of reading ≥ 40ppb | | Formaldehyde |
| SKC TD Tenax Sorbent Tubes | - | | TVOC |

**Table A-2**: Main differences and similarities between LEGO Serious Play (LSP) and Serious Brick Play (SBP)

| LSP core steps | LSP | SBP | SBP core steps |
| --- | --- | --- | --- |
| Challenge | Specialised/specific problem | Global Issues | **Read** |
| Construction | Individual build | Individual build | **Build** |
|  | Random bricks | Random bricks |  |
| Sharing | Group activity | Individual activity |  |
|  | Facilitators prompts questions | No prompt questions | **Describe** |
|  | Verbal description | Written description |  |
| Reflection | Verbal reflection | Post-test assessment | **Assess** |
| Duration | 2-8 hours | 40 minutes |  |

**Table A-3**: Results of ICC calculation for Inter-Rater Reliability. The same set of graders are used for all participants for a two-way random effects model based on a total score from individual graders to determine the absolute agreement.

| Round | ICC | 95% Confidence Interval | | F Test with True Value 0 | | | |
| --- | --- | --- | --- | --- | --- | --- | --- |
|  |  | Lower Bound | Upper Bound | Value | d*f* 1 | d*f* 2 | p-value |
| 1 | 0.79 | 0.72 | 0.83 | 6.2 | 251 | 1506 | <0.001* |
| 2 | 0.77 | 0.72 | 0.81 | 5.1 | 251 | 1506 | <0.001* |
| 3 | 0.8 | 0.76 | 0.83 | 5.6 | 241 | 1446 | <0.001* |

**Table A-4:** Linear mixed regression model testing the effect of experimental parameters on creativity scores. Participants are treated as random intercept and scaled week as random slopes. *Denotes statistical significance for p-value. Bolded column indicates the chosen model as the best fitted model for the data.

|  | Model Parameters | Model 0 | Model 1 | **Model 2** | Model 3 |
| --- | --- | --- | --- | --- | --- |
| Scaled Week | Standardised estimate | -0.06 | -0.08 | **-0.07** | -0.09 |
|  | 95% standardised CI | [-0.15 – 0.02] | [-0.17 – 0.00] | **[-0.16 – 0.01]** | [-0.18 – 0.00] |
|  | p-value | 0.146 | 0.063 | **0.101** | 0.056 |
|  | Effect size | 0.14 | 0.18 | **0.16** | 0.17 |
| Fungi | Standardised estimate | 0.05 | 0.08 | **0.07** | 0.06 |
|  | 95% standardised CI | [-0.03 – 0.14] | [-0.01 – 0.18] | **[-0.01 – 0.16]** | [-0.03 – 0.14] |
|  | p-value | 0.23 | 0.074 | **0.087** | 0.203 |
|  | Effect size | 0.06 | 0.09 | **0.08** | 0.06 |
| Bacteria | Standardised estimate | -0.02 | -0.02 | **0.01** | 0 |
|  | 95% standardised CI | [-0.11 – 0.07] | [-0.11 – 0.06] | **[-0.07 – 0.10]** | [-0.09 – 0.08] |
|  | p-value | 0.664 | 0.606 | **0.765** | 0.924 |
|  | Effect size | 0.02 | 0.02 | **0.01** | 0 |
| Formaldehyde | Standardised estimate | -0.09 | -0.09 | **-0.09** | -0.1 |
|  | 95% standardised CI | [-0.18 – 0.00] | [-0.19 – 0.00] | **[-0.18 – 0.01]** | [-0.19 – -0.00] |
|  | p-value | 0.059 | 0.051 | **0.066** | 0.044* |
|  | Effect size | 0.09 | 0.09 | **0.09** | 0.1 |
| CO | Standardised estimate | 0.01 | 0.01 | **0.04** | 0.04 |
|  | 95% standardised CI | [-0.08 – 0.09] | [-0.08 – 0.09] | **[-0.04 – 0.12]** | [-0.05 – 0.13] |
|  | p-value | 0.865 | 0.869 | **0.355** | 0.408 |
|  | Effect size | 0.01 | 0.01 | **0.04** | 0.04 |
| PM_2.5_ | Standardised estimate |  | -0.08 |  |  |
|  | 95% standardised CI |  | [-0.16 – 0.01] |  |  |
|  | p-value |  | 0.069 |  |  |
|  | Effect size |  | 0.09 |  |  |
| TVOC | Standardised estimate |  |  | **-0.16** |  |
|  | 95% standardised CI |  |  | **[-0.24 – -0.08]** |  |
|  | p-value |  |  | **<0.001 ***** |  |
|  | Effect size |  |  | **0.20** |  |
| CO_2_ | Standardised estimate |  |  |  | -0.09 |
|  | 95% standardised CI |  |  |  | [-0.20 – 0.01] |
|  | p-value |  |  |  | 0.073 |
|  | Effect size |  |  |  | 0.08 |
| AIC | | 928.8 | 935.6 | **919.7** | 941.7 |
| AIC Weightage | | 0% | 0% | **100%** | 0% |
| Chi-sq comparison to base model | | | 3.32  p = .068 | **16.96**  **p < . 0001** | 3.21  p = .073 |

**Figure A-5:** Sensitivity analysis showing standardised coefficients and 95% confidence intervals for creativity associated with a one-unit increase in IAQ parameter of interest scores, adjusted for 0, 1 and 2 covariate IAQ parameters of interest. Coefficients adjusted for other environmental parameters (CO, Formaldehyde, Bacteria, Fungi), with participant ID as random intercepts and week (scaled and centred) as random slopes. (a) PM_2.5_ adjusted sequentially for inclusion of CO_2_, TVOC, and both CO_2_ and TVOC. There is a substantial reduction in the absolute value of the PM_2.5_ coefficient when TVOC is added to the model. (b) TVOC adjusted sequentially for inclusion of CO_2_, PM_2.5_, and both CO_2_ and PM_2.5_. There is no reduction in the absolute value of the TVOC coefficient when either CO_2_ or PM_2.5_ are added to the model. (c) CO_2_ adjusted sequentially for inclusion of PM_2.5_, TVOC, and both PM_2.5_ and TVOC. There is a substantial reduction in the absolute value of the CO_2_ coefficient when TVOC is added to the model.

| 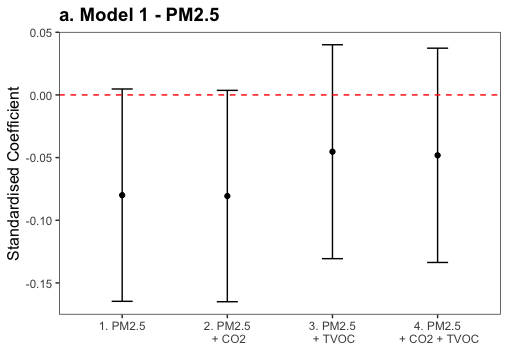 | 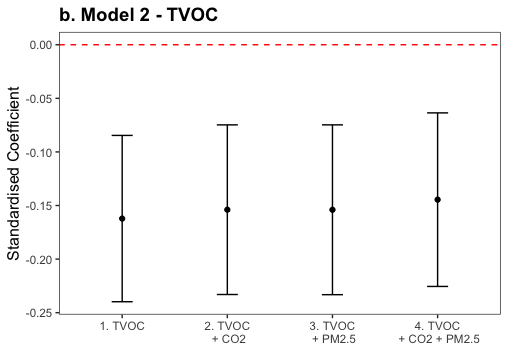 |
| --- | --- |
| 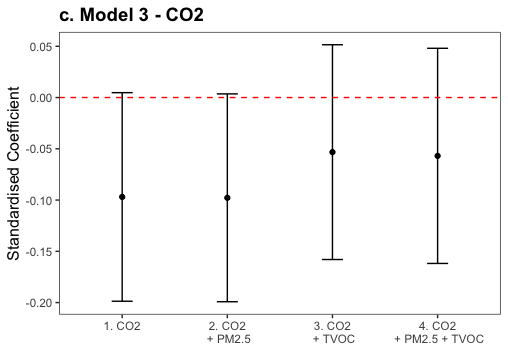 |  |

**Table A-6:** Correlation of SBP scores with Alternative Uses Task scores (n = 32)

|  | **SBP** | **AUT Components** | | | | **AUT Mean** |
| --- | --- | --- | --- | --- | --- | --- |
|  |  | Originality | Fluency | Flexibility | Elaboration |  |
| **SBP** | 1.00 | 0.09 | 0.27 | 0.32 | 0.47** | 0.42* |
| Originality | 0.09 | 1.00 | 0.31 | 0.45** | -0.11 | 0.30 |
| Fluency | 0.27 | 0.31 | 1.00 | 0.91*** | 0.21 | 0.94*** |
| Flexibility | 0.32 | 0.45** | 0.91*** | 1.00 | 0.09 | 0.88*** |
| Elaboration | 0.47** | -0.11 | 0.21 | 0.09 | 1.00 | 0.50** |
| **AUT Mean** | 0.42* | 0.30 | 0.94*** | 0.88*** | 0.50** | 1.00 |

* p < .05, ** p < .01, *** p < .001

***Table A-7:*** *Component matrix of Exploratory Factor Analysis of SBP and AUT constructs*

|  | **Component** | |
| --- | --- | --- |
|  | **1** | **2** |
| **SBP** |  | 0.437 |
| **Originality** | 0.470* |  |
| **Fluency** | 0.902* |  |
| **Flexibility** | 1.003* |  |
| **Elaboration** |  | 1.000 |

Estimation method: Maximum Likelihood, rotation method: Geomin Oblique. * p < .1

***Supplementary information A-8:***

We tested the convergent and discriminant validity of the SBP method by taking a group of 32 participants and asking them to complete both the first SBP task as well as two Alternative Uses Task (AUT) trials. AUT responses were graded by three members of the research team. AUT scores for the two trials were averaged for each participant. Pearson correlation scores were calculated for SBP with the component constructs of AUT, as well as the overall mean AUT score (Table A-6). In general, SBP showed low correlation with AUT constructs, indicating good discriminant validity, and low overlap in constructs. The elaboration component of AUT had the highest correlation with SBP (r = 0.47, p = .007) and there was a moderate correlation with the overall AUT score (r = 0.42, p = .017), indicating a possible convergence. We therefore ran an exploratory factor analysis on the SBP and AUT scores (Table A-7). This indicated a two-factor solution, with SBP and Elaboration in the second factor. This indicates good discriminant validity between SBP and three of the AUT components (Originality, Fluency, Flexibility). However, the factor loading of 0.437 for SBP is too low to indicate adequate convergent validity with the Elaboration component of AUT. This suggests that SBP measures a related but distinct creativity construct to AUT.

Appendix B: Challenges provided to participants

**Climate Change**


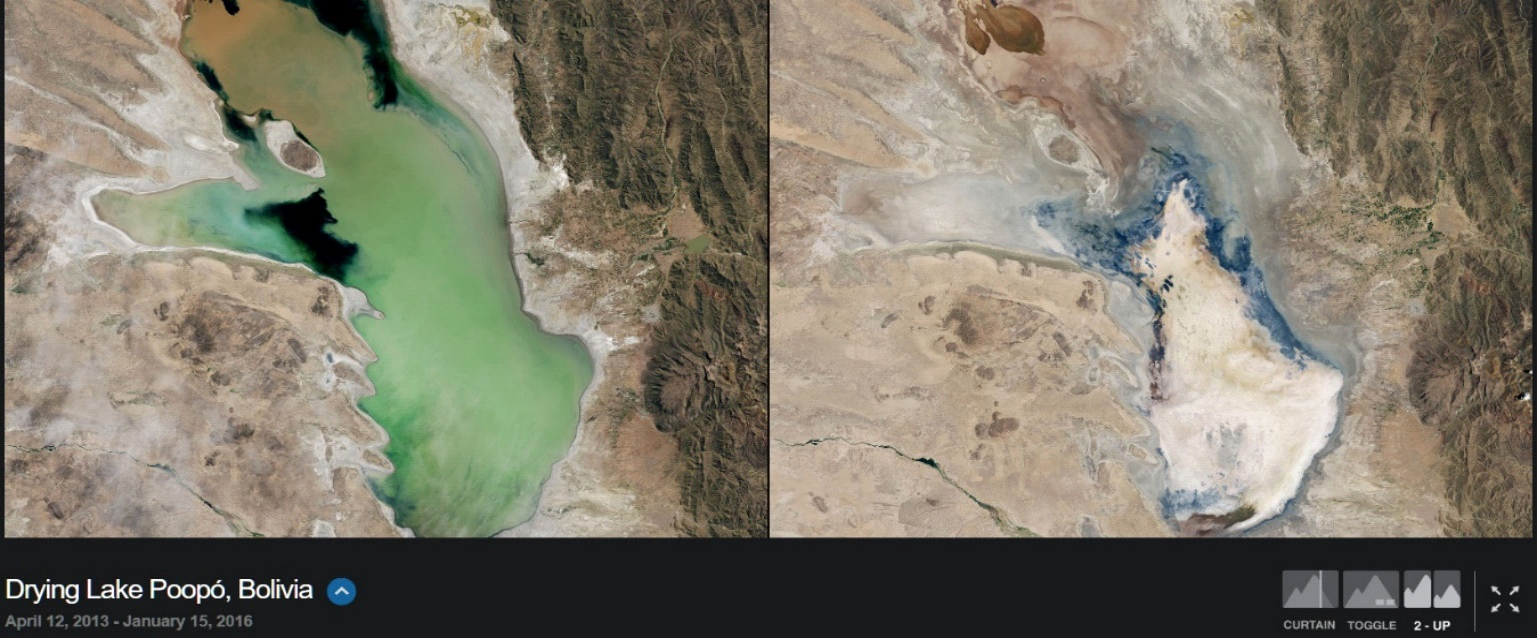

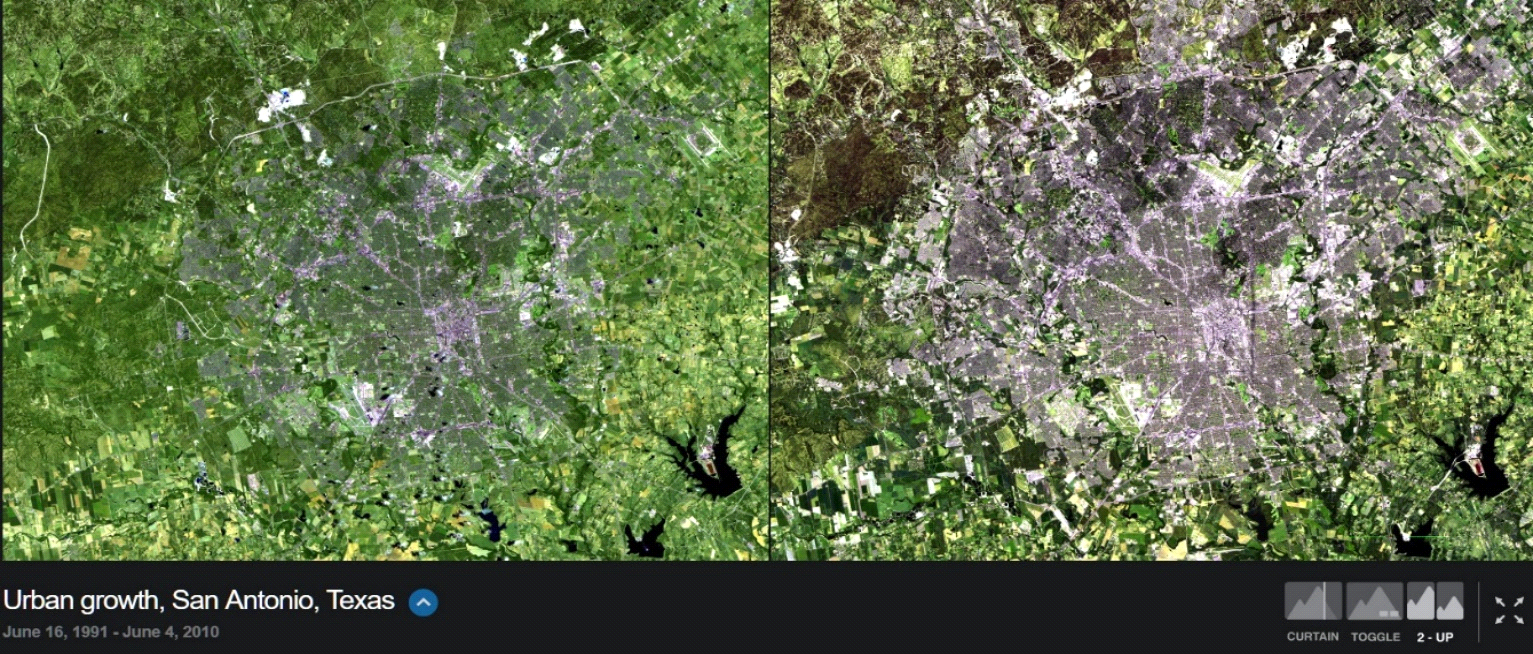
Source: NASA’s images of change (<https://climate.nasa.gov/images-of-change?>)

The images above representing different landscapes over a different part of the world were taken over different time frames (e.g. over the years or months). The enormous change seen in these images reveals the devastating outcomes of climate change due to human activity. The unmitigated greenhouse gas due to human activities has been anticipated to increase the global temperature from 2.6ºC to 4.8ºC by 2100. Greenhouses gases, such as carbon dioxide, trap heat in the atmosphere and regulate our climate. These gases exist naturally, but humans add more carbon dioxide by burning fossil fuels for energy (coal, oil, and natural gas) and by clearing forests. Greenhouse gases act like a blanket. The thicker the blanket, the warmer our planet becomes. At the same time, the Earth’s oceans are also absorbing some of this extra carbon dioxide, making them more acidic and less hospitable for sea life.

Humans and wild animals face new challenges for survival because of climate change. More frequent and intense drought, storms, heat waves, rising sea levels, melting glaciers and warming oceans can directly harm animals, destroy the places they live, and wreak havoc on people’s livelihoods and communities. As climate change worsens, dangerous weather events are becoming more frequent or severe. People in cities and towns are facing the consequences, from heat waves and wildfires to coastal storms and flooding.

As such, what could be done to slow down climate change?

You are required to build a model with the Lego bricks by expressing your ideas/thoughts on to this building challenge.

Upon building give a short description of your model below.

**References:**

- Ai-Lien, C. (2019). Singapore Budget 2019: Preparing for climate change over long term, making Singapore a global city and home for all. Stratis Times online. Singapore.
- NASA. "Blanket around the earth." Retrieved 18 April 2019, 2019, from <https://climate.nasa.gov/causes/>.
- WWF. "Effects of climate change." from [https://www.worldwildlife.org/threats/effects-of-climate-cha HYPERLINK "https://www.worldwildlife.org/threats/effects-of-climate-change"nge](https://www.worldwildlife.org/threats/effects-of-climate-change).
- (Ai-Lien 2019)(WWF)(NASA)

**Poverty**


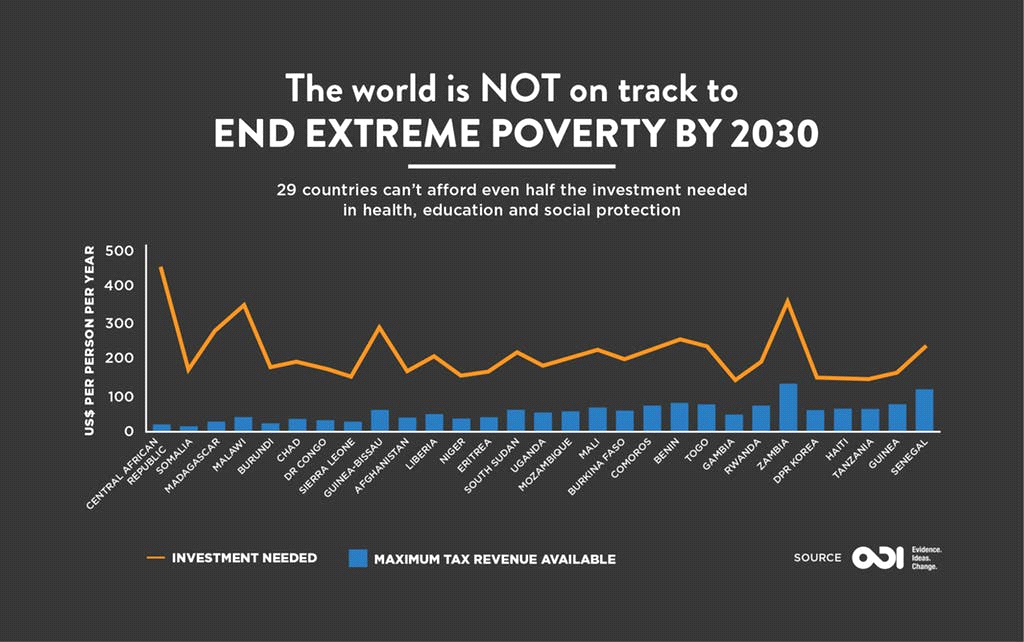


Poverty entails more than the lack of income and productive resources to ensure sustainable livings. Its manifestations include hunger and malnutrition, limited access to education and other basic services, social discrimination and exclusion, as well as the lack of participation in decision-making. According to the UN, the current statistics refers to more than 780 million people live below the international poverty line. 11% of the world population lives in extreme poverty. By 2030, 167 million children will live in extreme poverty if nothing is done.

Singapore, known as one of the wealthiest countries in the World with a GNI per capita of US$90, 570 as of November 2018 is also one of the countries in the world with large income gap. Recent statistics reveal that 1 in 10 Singaporeans are unable to meet basic needs in the form of food, clothing, shelter and other essentials. The top 20% of earners saw their real wages rise by 27% where the bottom 20% of earners saw their real wages fall by 8% between 1998 and 2010.

While the government tries to implement policies forcing on poverty, it is also essential to understand the complexities of poverty. Children from low-income families are more likely to experience developmental delays and learning difficulties compared to their peers. They are also more likely to experience anxiety, depression and problems with impulse control. Research had shown that the impact of the toxic stress of poverty on children’s brain development, which sometimes continues into adulthood. Employment assistance programmes may help with money and give them the confidence to secure a job, but will not eradicate this scourge of poverty.

As such, what could be done to change/improve the state of poverty?

You are required to build a model with the Lego bricks provided by expressing your ideas/solutions/thoughts about this building challenge.

Upon building give a short description of your model below.

**References:**

- Brown, K. (2019). "6 Global Issues to watch in 2019." Retrieved 26 April, 2019, from <https://unfoundation.org/blog/post/6-global-issues-to-watch-2019/>.
- Ng, C. (2018). Commentary:This is what the face of poverty looks like. Channelnewasia. Singapore/Wedsite.
- *Goal 1: End Poverty in all its forms everywhere*. (n.d.). Retrieved from Sustainable Development Goals: 17 Goals to Transform Our World: <http://www.un.org/sustainabledevelopment/poverty>
- Estimation derived from (2012). *Key Household Income Trends.* Singapore: Department of Statistics Singapore. Retrieved from Singaporeans Against Poverty: sgagainstpoverty.org
- Economic Growth and Inequality in Singapore: The Case for a Minimum Wage”, *International Labour Review*, Vol 152, No. 1, (2013), pp. 107-123.Ibid
- (Ng 2018, Brown 2019)

**Mental Health**


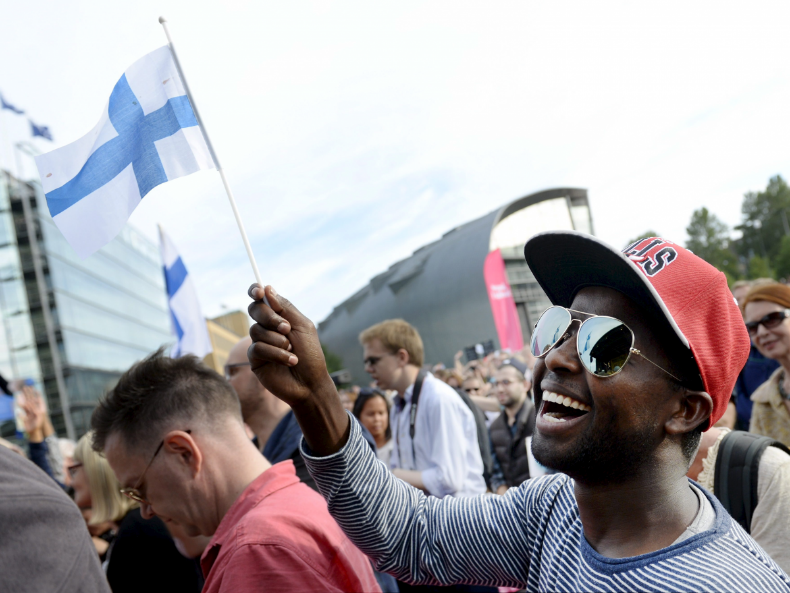
Finland is rated one of the happiest countries on earth, according to the World Happiness Index Report, 2018. However, mental health statistics depict a different scenario as shown below.


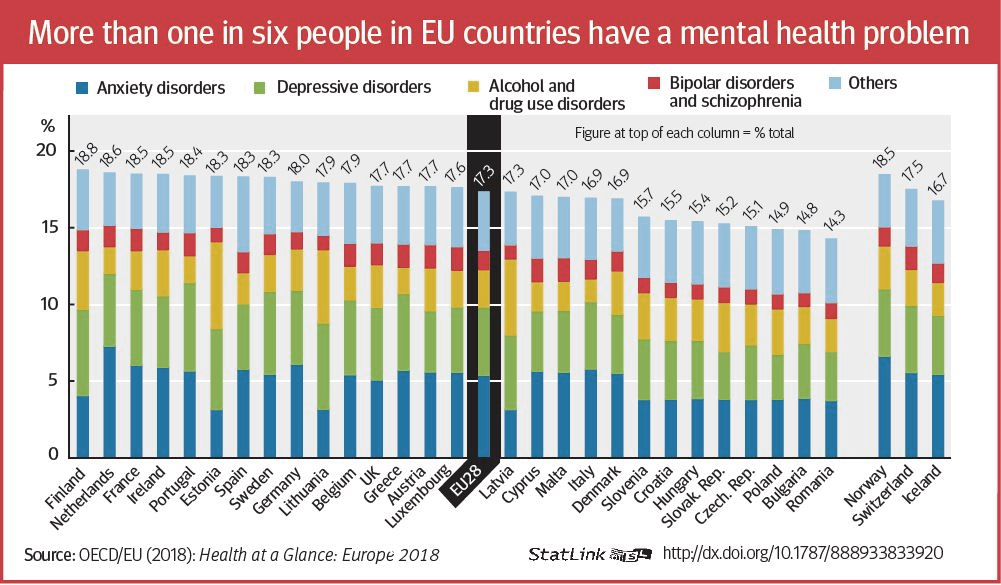


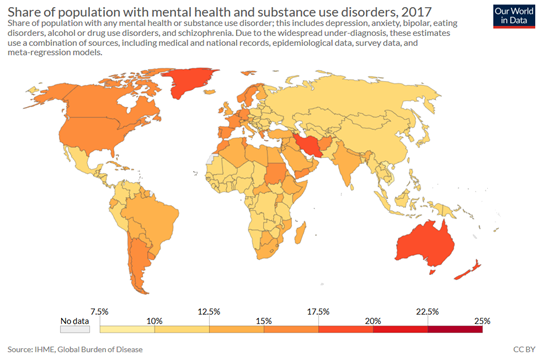

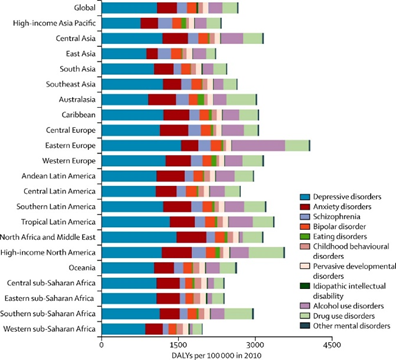


Mental health and behavioural problems (e.g. depression, anxiety and drug use) are reported to be the primary drivers of disability worldwide, causing over 40 million years of disability in 20 to 29-year-olds. Major depression is thought to be the second leading cause of disability worldwide and a significant contributor to the burden of suicide and ischemic heart disease. Mental health is the new global crisis that is a growing public health concern where in 2018 the WHO (World Health Organisation) Director-General identified mental health for accelerated implementation within WHO’s work. It is estimated that 970 million people worldwide had a mental or substance use disorder in 2017.

From the chart on the left shows that globally, mental and substance use disorders (comprises a range of disorders including depression, anxiety, bipolar, eating disorders, schizophrenia, intellectual developmental disability, and alcohol and drug use disorders) are very common: around 1-in-6 people (15-20%) have one or more mental or substance use disorders.

The breakdown of the disorders shown on the chart (right), had used data from the Global Burden of Diseases, Injuries, and Risk Factors Study 2010 to estimate the burden of disease attributable to mental and substance use disorders in terms of disability-adjusted life years (DALYs). Among all continents, South East Asia, depressive disorders are one of the highest.

In Singapore, a recent study done in 2016 found that 1 in 7 people in Singapore (13.9 per cent) have experienced specific mood, anxiety, or alcohol use disorders in their lifetime whereas in EU it is estimated that 1 in every 6 people experience a mental health problem. This proportion is an increase from roughly one in eight (12 per cent) six years prior.

Mental health is a major concern when it comes to well-being as such what could be done to change/improve this state?

You are required to build a model with the Lego bricks provided by expressing your ideas/solutions/thoughts about this building challenge.

Upon building give a short description of your model below.

**References**

- . "Mental health statistics: UK and worldwide." 2019, from <https://www.mentalhealth.org.uk/statistics/mental-health-statistics-uk-and-worldwide>.
- Cornford, E. H. a. K. (2018). "Finland'd mental health challenge." Retrieved May, 2019, from <http://oecdobserver.org/news/fullstory.php/aid/6164/Finland_92s_mental_health_challenge.html>.
- Lim, S. (2018). "1 in 7 people in Singapore have experienced a mental disorder – and some millennials are more susceptible to mood and alcohol-related ones." Retrieved May, 2019, from <https://www.businessinsider.sg/1-in-7-people-in-singapore-have-experienced-a-mental-disorder-and-some-millennials-are-more-susceptible-to-mood-and-alcohol-related-ones/>.
- Organisation, W. H. (2019). Special initiative for mental health (2019-2023).
- Roser, H. R. a. M. (2018) Mental Health. Our World in Data
- Whiteford, H. A., et al. (2013). "Global burden of disease attributable to mental and substance use disorders: findings from the Global Burden of Disease Study 2010." The lancet **382**(9904): 1575-1586.
- (Whiteford, Degenhardt et al. 2013, Cornford 2018, Lim 2018, Roser 2018, Organisation 2019)

**Ageing Population**

Virtually every country in the World is experiencing growth in the number and proportion of older persons in their population. Globally, the number of persons aged 80 or over is projected to triple by 2050, from 137 million in 2017 to 425 million in 2050. By 2100 it is expected to increase to 909 million, nearly seven times its value in 2017. As many such countries are likely to face fiscal and political pressures about public systems of health care, pensions and social protections for a growing older population.


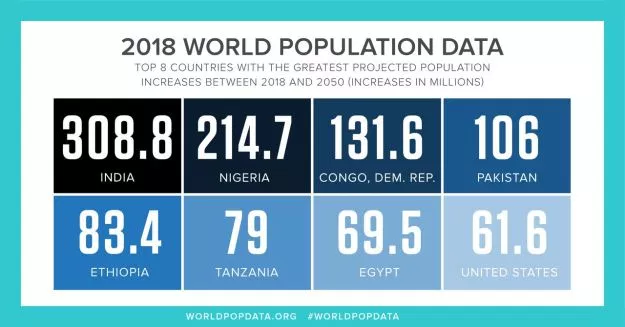


From the statistics above, these top 8 countries are projected to have an increasing population. **Nigeria will become the third most populous country as its population rises to 411 million,** up 109% from 2018. Nigeria is currently the seventh most populous country.


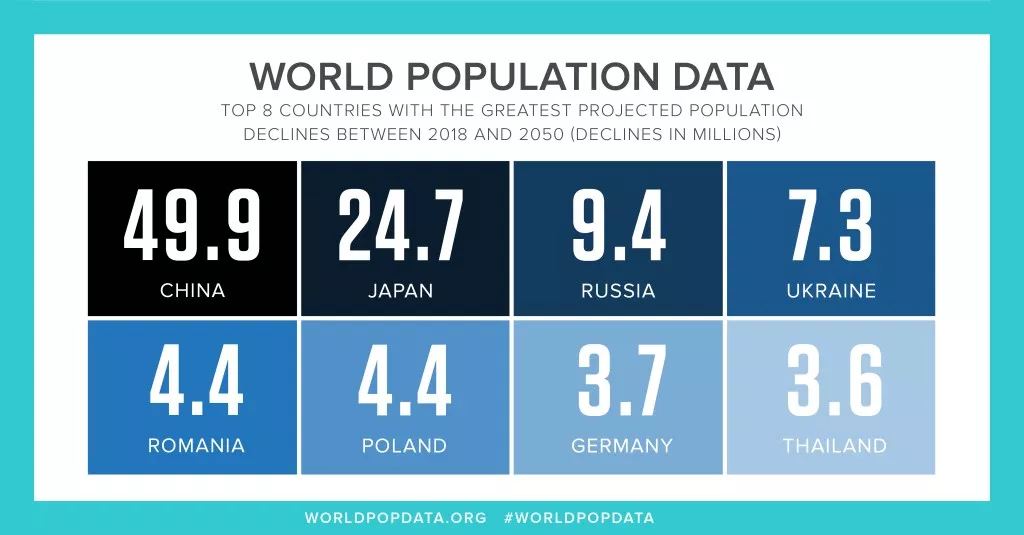


Top 8 countries listed in the table above are projected for the decline in population by 2050. **A total of 38 countries will have smaller populations in 2050 than in 2018.** China will register the most substantial numerical population decrease, about 50 million followed by Japan at 25 million and Russia at 9.4 million. Romania will see the most significant percentage decline in population (23 %). **China’s population will decrease by about 50 million from its current size to 1.34 billion.** India will supplant China as the world’s most populous country with 1.68 billion people.

The size and age composition of a population is determined jointly by three demographic processes: fertility, mortality and migration. Declining fertility and increasing longevity are the key drivers of population ageing globally; international migration has also contributed to changing population age structures in some countries and regions.

For the past 11 years in Singapore, the growth rates among the population have been more stagnant than ever. As shown in the figure below, total population growth in 2018 was 0.5% (compared to 0.1% in the previous year). Resident growth has remained stable since 2012.


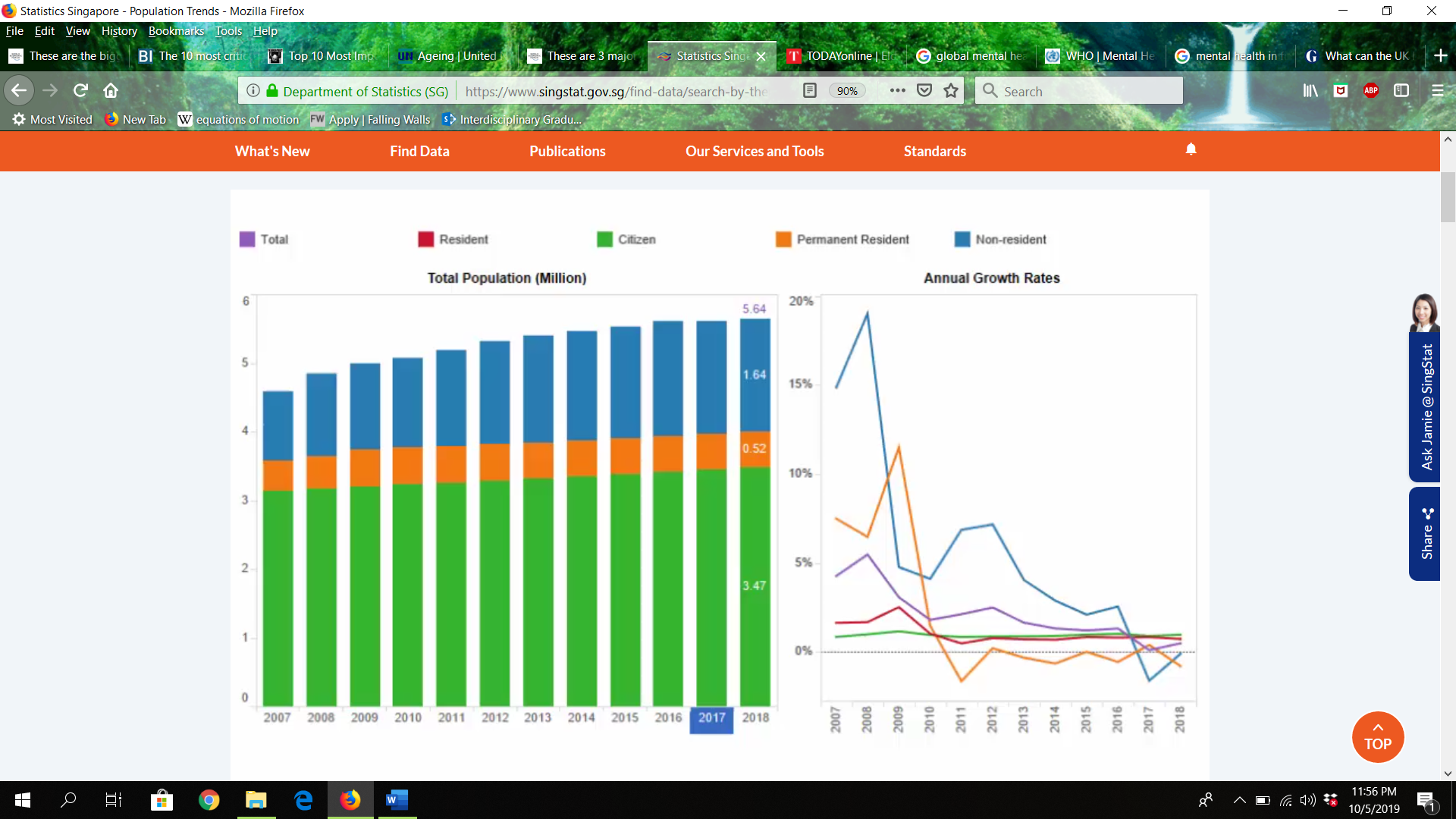
 Singapore population demographics over 11 years

As projected that life expectancy of Singapore population will rise further due technology advances. The life expectancies for men and women born in Singapore between 2010 and 2015 will be 80.1 and 84.5, respectively. These would increase to 85.6 and 89.3 years respectively for those born between 2045 and 2050. The median age of the Singapore population would climb from 40 years in 2015 to 47 years in 2030 and 52.8 years in 2050. The break down of the males and females according to their ages as shown in the image below shows a clearer picture of the ageing crisis in Singapore.


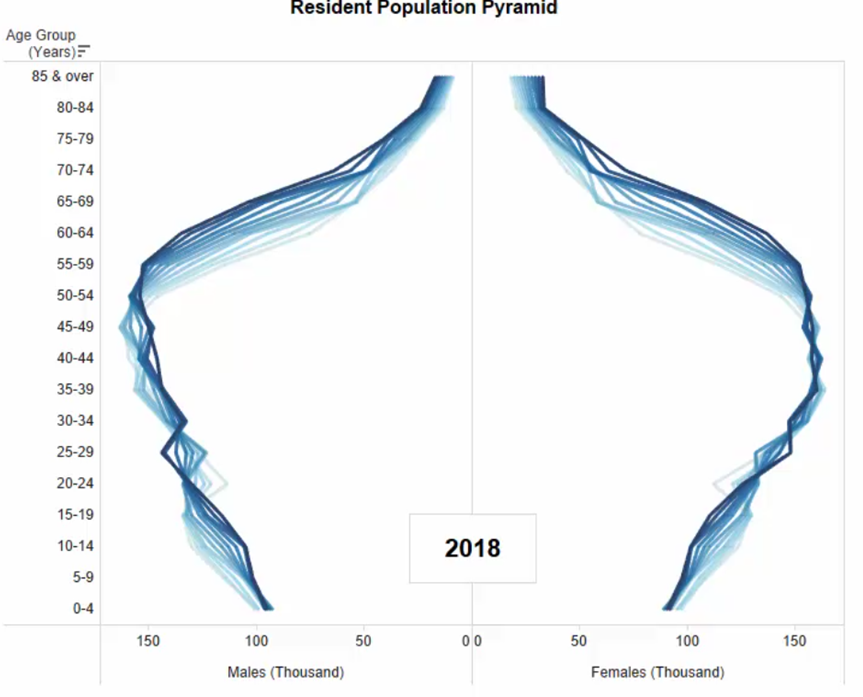


This would mean that the dependency ratio will be halved to almost 1:1, with one adult supporting a child or an older adult. In 2015, there were 100 adults – persons aged 20 to 64 years – supporting about 50 children and elderly persons. However, by 2050, 100 adults would have to support about 95 children and elderly persons.

Ageing and the resources strain that comes along with it is a concern prevailing for ages, as such what could be done to change/improve this state?

You are required to build a model with the Lego bricks provided by expressing your ideas/solutions/thoughts about this building challenge.

Upon building give a short description of your model below.

**References**

- EN, S. M. (2017). Elderly to make up almost half of S’pore population by 2050: United Nations. Today Singapore.
- Singapore, G. o. (2019). Population Overview. S. Singapore.
- Toshiko Kaneda, C. G., Kaitlyn Patierno (2018). 2018 World Population Data Sheet With Focus on Changing Age Structures. P. R. Bureau. <https://www.prb.org/2018-world-population-data-sheet-with-focus-on-changing-age-structures/>.
- (EN 2017, Toshiko Kaneda 2018, Singapore 2019)

**Air Pollution**

As economies and carbon footprints grow, urbanisation accelerates, and global temperatures rise, our air quality deteriorates. Generally termed as climate change. Pollution may seem like an environmental problem, but, according to the World Health Organisation (WHO), there are severe health implications. As of 15^th^ May 2019, Mexico City had declared an environmental emergency due to forest fires. The visual severity is shown in the image below.


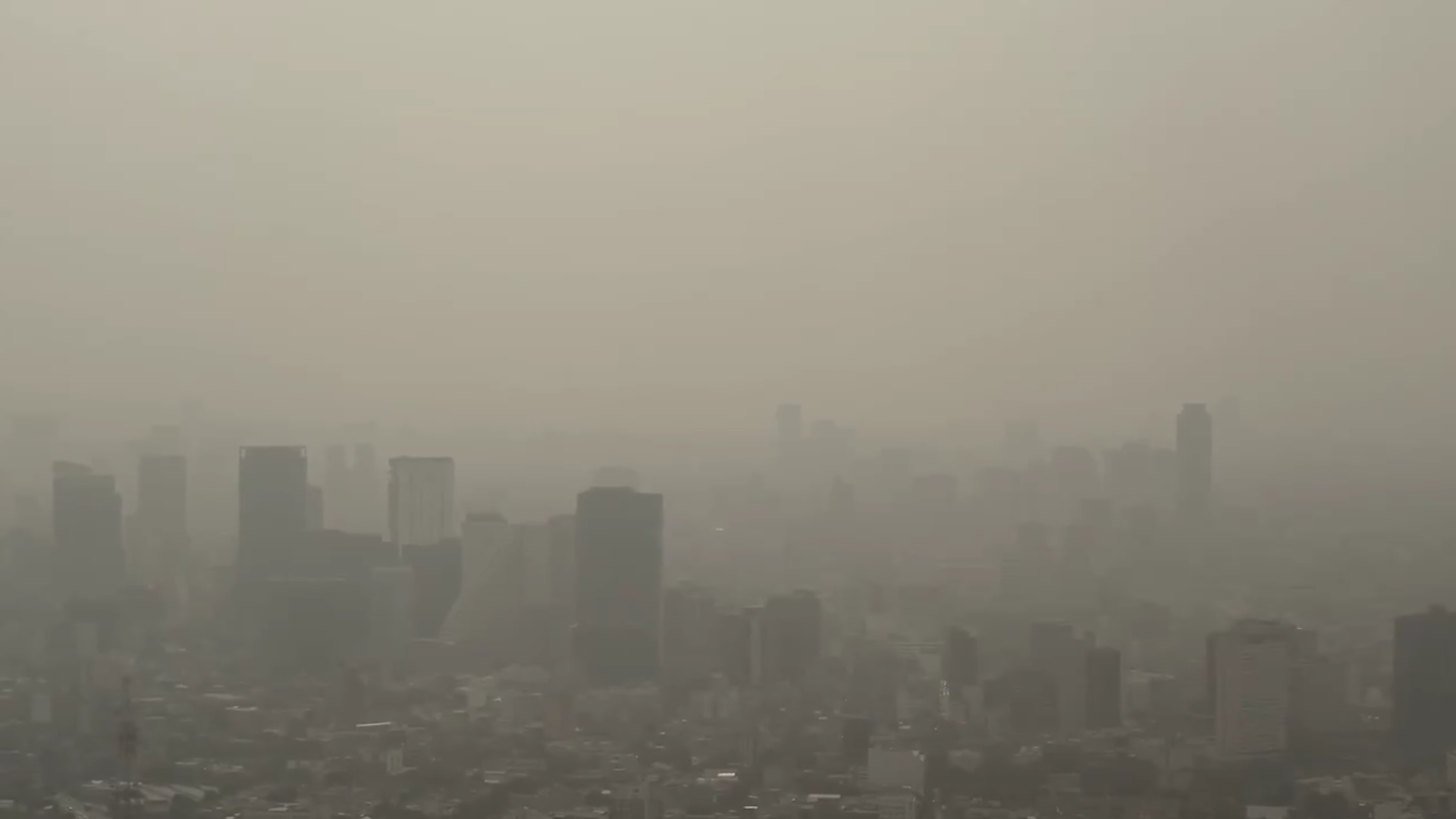
Source: Mexico from a drone, tweet from Santiago Arau

Each year, more than 400,000 Europeans die prematurely as a consequence of poor air quality, and many more suffer from respiratory and cardiovascular diseases caused by air pollution. Around 7 million people die every year from exposure to fine particles in polluted air that penetrate deep into the lungs and cardiovascular system, causing diseases including stroke, heart disease, lung cancer, chronic obstructive pulmonary diseases and respiratory infections, including pneumonia. In economic terms, bad air quality costs over €20bn a year to the European economy, due to increased medical costs and reduced worker productivity.

Ambient air pollution alone caused some 4.2 million deaths in 2016, while household air pollution from cooking with polluting fuels and technologies caused an estimated 3.8 million deaths in the same period. More than 90% of air pollution-related deaths occur in low- and middle-income countries, mainly in Asia and Africa as shown in the image below, followed by low- and middle-income countries of the Eastern Mediterranean region, Europe and America.


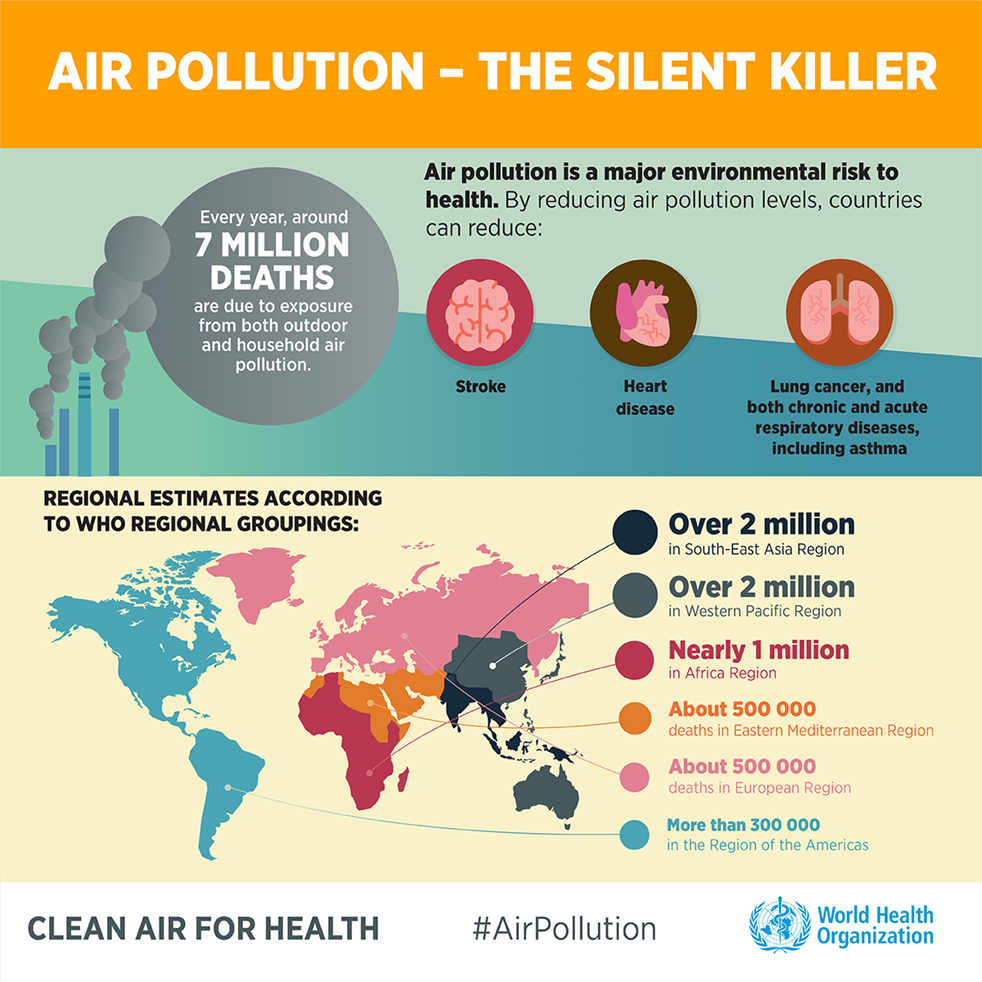


WHO recognises that air pollution is a critical risk factor for noncommunicable diseases (NCDs), causing an estimated one-quarter (24%) of all adult deaths from heart disease, 25% from stroke, 43% from chronic obstructive pulmonary disease and 29% from lung cancer.

Indoor air pollution is one of the world's most significant environmental problems. Based on figures from the Institute for Health Metrics and Evaluation (IHME), 2.6 million people died prematurely in 2016 from illness attributable to household air pollution. It is predominantly women and young children who are killed by indoor air pollution.


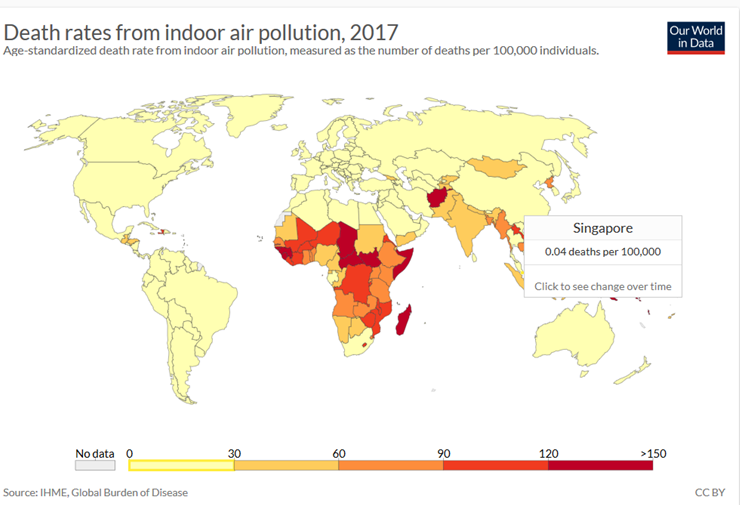


The chart below shows death rates from indoor air pollution plotted against rates from outdoor particulate air pollution. Countries which lie above this line have higher death rates from indoor pollution relative to outdoor. Here we see that for a large number of countries, indoor air pollution is still dominant.


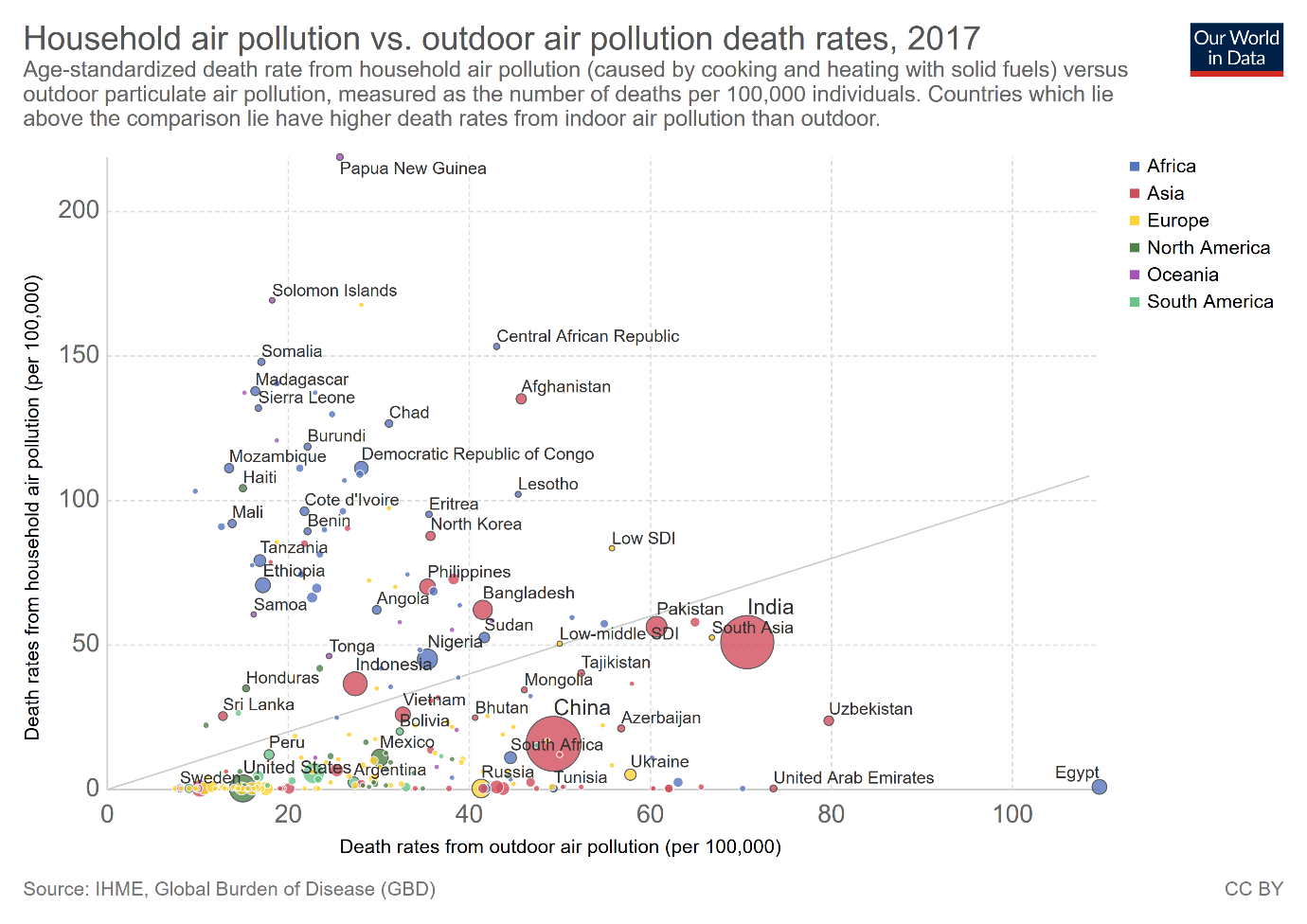


Under the blanket for climate change, air pollution has a detrimental effect on our health without us knowing it. As such, what could be done to change/improve this state?

You are required to build a model with the lego bricks provided by expressing your ideas/solutions/thoughts about this building challenge.

Upon building give a short description of your model below.

**References**

- Dodson, K. (2018). Why WHO’s First Conference on Air Pollution Matters for Health and Climate. U. N. Foundation.
- Europa, S. (2018). "How serious is air pollution?" Environment and Sustainability News. from <https://www.scitecheuropa.eu/serious-air-pollution/83486/>.
- News, B. (2019). Mexico City pollution: Residents urged to stay indoors.
- Ritchie, M. R. a. H. (2019). "Indoor Air pollution." Our World in Data.
- WHO Public health, environmental and social determinants of health (PHE)
- (WHO , Dodson 2018, Europa 2018, News 2019, Ritchie 2019)

**Biodiversity**

Biodiversity is under serious threat as a result of human activities population growth and resource consumption, climate change and global warming, habitat conversion and urbanisation, over-exploitation of natural resources and environmental degradation. UN scientists warn that roughly 1 million plant and animal species are on the verge of extinction due to human activity. It would be the first mass extinction since humans started walking the earth and have dire implications for the survival of our species.

Most of our planet is covered in water. We depend on the oceans to maintain our rainwater system and many populations rely on it for food and income. Oceans also absorb carbon dioxide and produce about 30% of our oxygen. Despite its importance, the ocean is under threat. Overfishing and unsustainable fishing practices are causing the endangerment and extinction of many fish species. Species began going extinct at a much faster pace. Over the past five centuries, researchers have recorded 514 animal extinctions on land. Before 1500, a few species of seabirds are known to have vanished. Since then, scientists have documented only 15 ocean extinctions, including animals such as the Caribbean monk seal and the Steller’s sea cow. While these figures are likely underestimated.


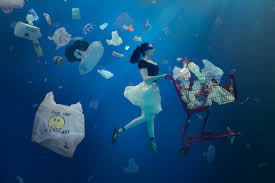


The pictures show the reliance and the number of pollutants we produce every day to sustain our survival. One-time use waste being irresponsibly bumped into the oceans in the form of disposal is one of the main reasons for escalating extinction in recent year, as shown in the picture below.


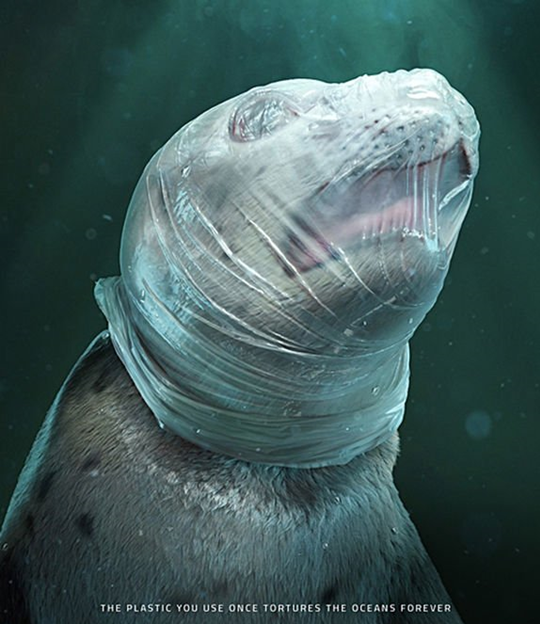


The table below provides a list of country that produces a high volume of plastic waste. Which also means how much of such non-biodegradable products we produce every day to meet the growing population needs.


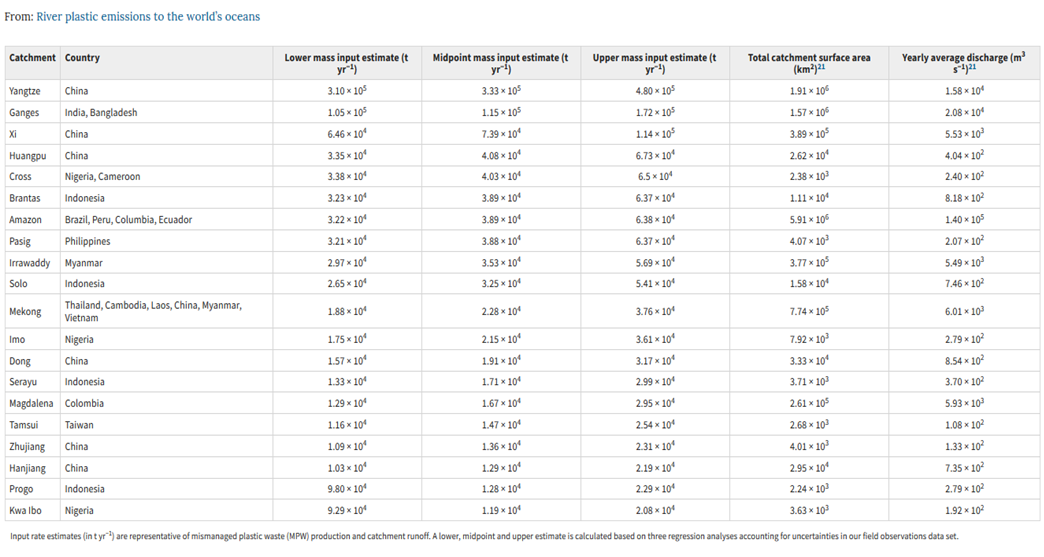


A study in California was the first to report levels of micro-plastics in river surface waters, with sampling in Los Angeles River, San Gabriel River and tributary Coyote Creek. The report found substantial temporal variations in plastic contamination levels. For a given location, the study found up to three orders of magnitude differences between plastic concentrations measured at different periods. These variations were mostly explained by events of dry and wet weather, implying that runoff plays an essential role in the transport of plastics into freshwater systems. In recent years, more studies sampled plastic in surface waters of rivers. In Europe, studies estimated that the Danube River releases 530–1,500 tonnes of plastic into the Black Sea annually. Another European study estimated that 20–31 tonnes flow into the North Sea every year from the Rhine River, with different locations along this river demonstrating the presence of significant sources (for example, wastewater treatment plants, tributaries) and sinks. In the Italian Po River, sampled concentrations differed by one order of magnitude between winter and spring, emphasising seasonality of freshwater contamination in rivers.


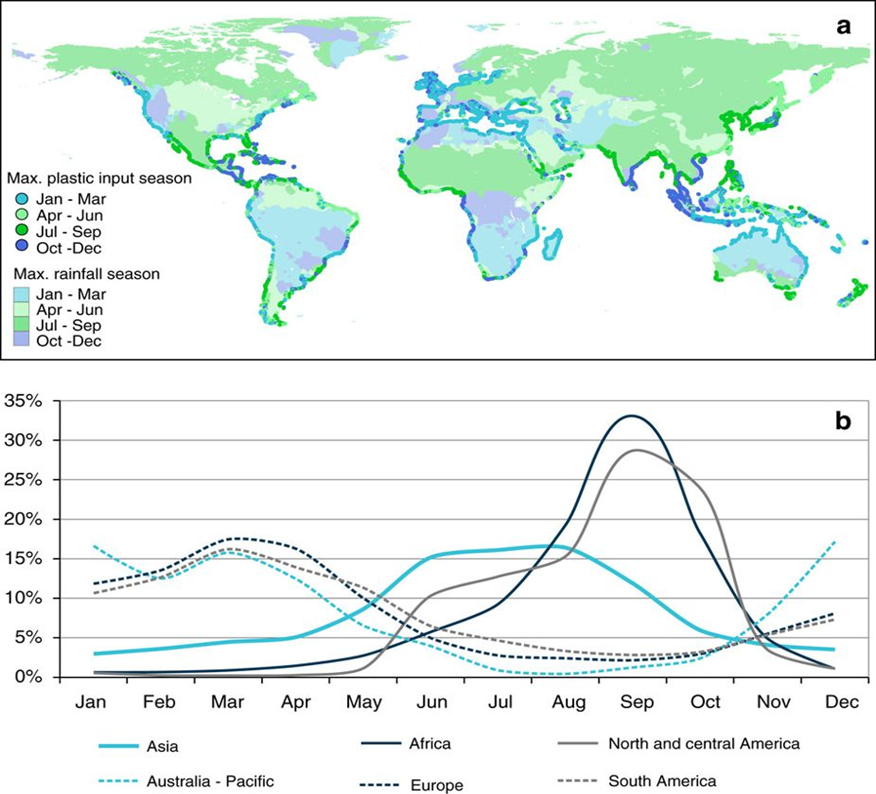


a) River outflow locations are indicated by trimester period when respective peak input occurs. Peak seasons for precipitation rates from GLDAS are mapped on the continental landmass, showing a clear correlation with our predicted inputs. (b) Relative seasonality of plastic inputs from rivers into the ocean by continents. Continental contributions are expressed in percentage of respective annual mass inputs.

Global estimate of plastic emissions from rivers into the world’s oceans: between 1.15 and 2.41 million tonnes per year. Most of this river plastic input is coming from Asia, which emphasises the need to focus on monitoring and mitigation efforts in Asian countries with rapid economic development and poor waste management. Research on freshwater plastic pollution is a relatively new field, and most efforts have been carried out in industrialised countries of Europe and North America. While many indicators suggest a dominant contribution of plastics from Asian countries, there is very little data to document these assumptions and thoroughly verify the validity of our model. The relatively high concentrations of ocean plastic found at the surface of the North Pacific Ocean where buoyant plastics originating from Asia suggests that our assumptions are plausible.

The impact of human activities had caused a detrimental effect on biodiversity more significantly with plastics and its usages. As such, what could be done to change/improve this state?

You are required to build a model with the lego bricks provided by expressing your ideas/solutions/thoughts about this building challenge.

Upon building give a short description of your model below.

**References**

- Laurent C. M. Lebreton, J. v. d. Z., Jan-Willem Damsteeg, Boyan Slat, Anthony Andrady & Julia Reisser (2017). "River plastic emissions to the world’s oceans." Nature Communications.
- Wernick, A. (2019). A UN report says Earth faces 'unprecedented' threat to biodiversity. Living on Earth - PRI.
- Zimmer, C. (2015). Ocean Life Faces Mass Extinction, Broad Study Says. The New York Times.
- (Zimmer 2015, Laurent C. M. Lebreton 2017, Wernick 2019)

Appendix C: List of usual Uses

**Climate Change**

| Solar panels |
| --- |
| Windmill |
| 3 Rs (Reuse, Reduce, Recycle) |
| Policy |
| Industrial Uses |
| Education |
| Trees |
| Plantation |
| Green building |
| Tidal energy |
| Vehicles |

**Poverty**

| Basic Metaphor (e.g Bridges which connect the poor and rich) |
| --- |
| Homeless people |
| Schools |
| Policy |
| Money |
| Houses |
| Education |

**Mental Health**

| Companion robots |
| --- |
| Companion |
| Education |
| Counsellors |
| Hospital/Clinics |

**Ageing Population**

| Committee Centres |
| --- |
| Policy |
| Elderly Care |
| Education |
| Elderly friendly amenities |
| Facilities |

**Air Pollution**

| Air purifier |
| --- |
| Solar panels |
| Windmill |
| 3 Rs (Reuse, Reduce, Recycle) |
| Trees |
| Plantation |
| Green building |
| Vehicles |
| Filters |
| Cooking fuels |
| Education |
| Policy |
| Industrial Uses |

**Biodiversity**

| 3 Rs (Reuse, Reduce, Recycle) |
| --- |
| Plastic pollution |
| Collecting sea plastic |
| Policy |
| Education |

*Summary of Serious Brick Play (SBP) methodology*


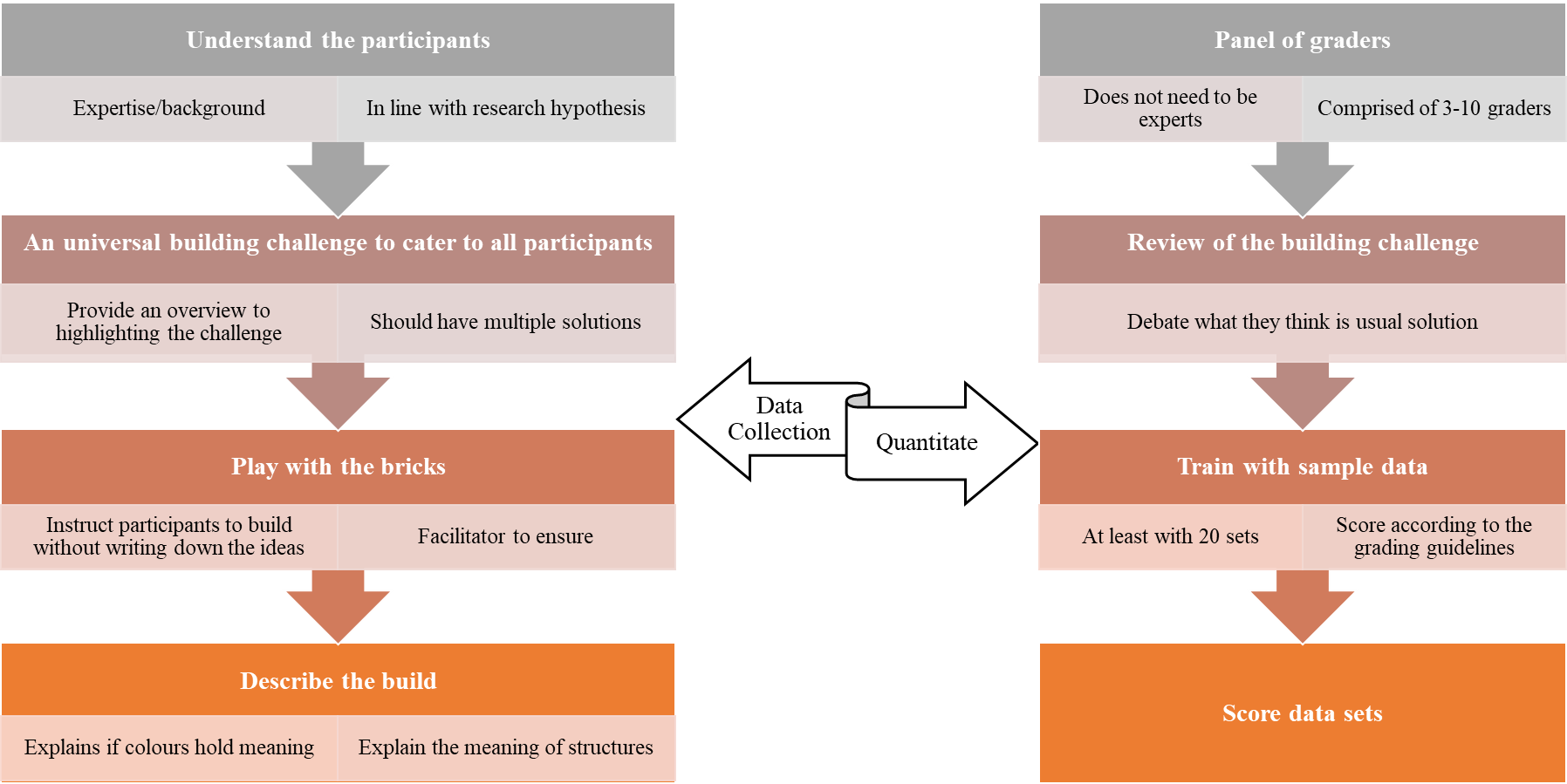


| Appendix D: Scoring Rubrics  **Point 1: Originality** | |  |  |  |  |  |  |
| --- | --- | --- | --- | --- | --- | --- | --- |
|  | **Usual Uses** | | | **Unusual Uses** | | | |
| **Score** | 1 | | | 2 | | | |
| Please refer to the 'Usual Uses' tab to find out the usual uses of the individual scenario | | | | | | |  |
|  |  |  |  |  |  |  |  |
| **Point 2: Fluency** | |  |  |  |  |  |  |
|  | **Elaboration** | **No Elaboration** |  | *Plagiarism, -1 | |  |  |
| **Score** | 1 | 0 |  |  |  |  |  |
| Please make the description of the model follows the following guidelines before scoring, | | | | | | | |
| 1. Description is being link back to the scenario |  |  |  |  |  |  |  |
| 2. Description of the lego model built  (Description must be at least 70% done for the model; Description being link to the model) | | | | | | | |
| 3. Explanation of the lego model built (e.g. When/Where/How does the model is being utilise; Feasibility; Specificity) | | | | | | |  |
|  |  |  |  |  |  |  |  |
| **Point 3: Build** | |  |  |  |  |  |  |
|  | **Sophisticated Build** | **Normal Build** | **No Build** |  | *Plagiarism, -1 | |  |
| **Score** | 2 | 1 | 0 |  |  |  |  |
| Please refer to the following guidelines, | | |  |  |  |  |  |
| 1. Aesthetic; Colour coordination | | |  |  |  |  |  |
| 2. Creative usage of the lego bricks | | |  |  |  |  |  |
| 3. Amount of effort | |  |  |  |  |  |  |
| 4. Complexity |  |  |  |  |  |  |  |

Appendix E: Example solutions from two anonymised participants

**Round 3, Week 2, Participant X1**

*Building challenge: Poverty*

|  | Grader 1 | Grader 2 | Grader 3 | Grader 4 | Grader 5 | Grader 6 | Grader 7 | AVE |
| --- | --- | --- | --- | --- | --- | --- | --- | --- |
|  | 4 | 4 | 3 | 4 | 3 | 2 | 5 | 3.6 |

One of the problem faced by the poverty is that they do not have the access to clean water, that’s why I have designed a mobile water desalination plant. Basically the desalination plant was brought onto the ship, and it will travel to the poverty area to give out the clean water, so the people living in the poverty area will have the access to the clean water.

First, sea water will be brought up from the grey unit from the bottom end part of the ship(below the black turbine), then the water will be transported to the middle part of the ship, which is the desalination and filtration parts (the two black circular unit at the middle), the clean water will be stored in the tank located on top of the two black circular unit. After that the ship will travel to the poverty area and clean water will be released from the tank (from the top white tank to the pink color parts of the ship).

| 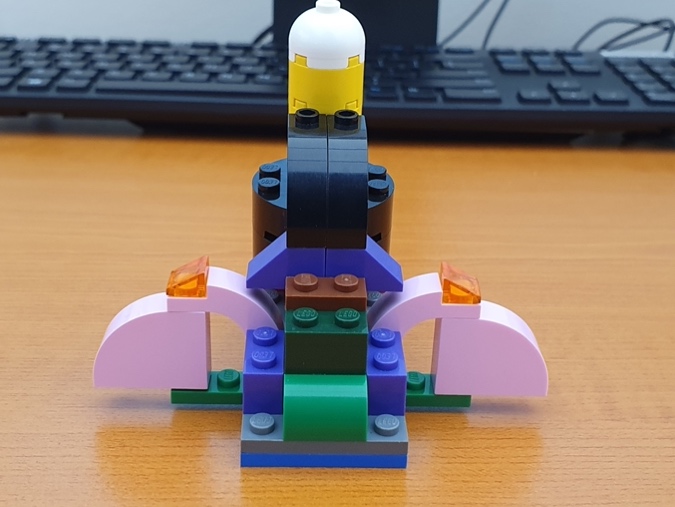 | 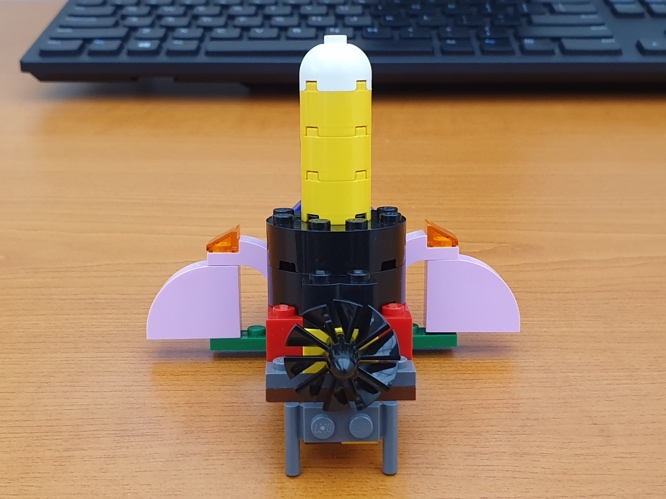 |
| --- | --- |
| 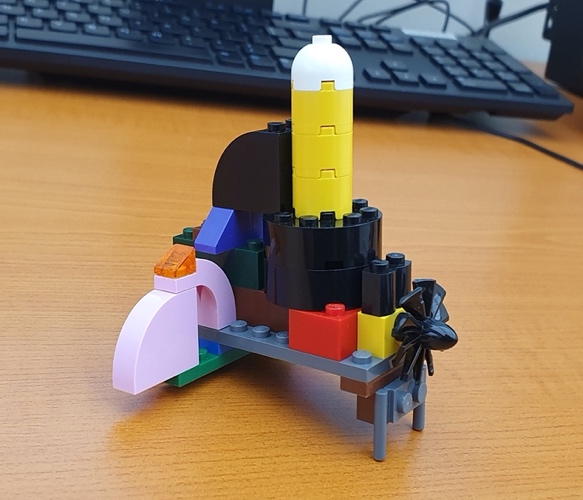 | 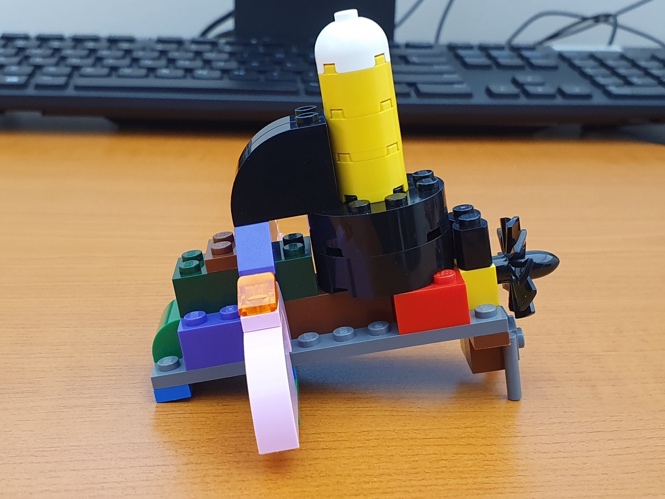 |
| 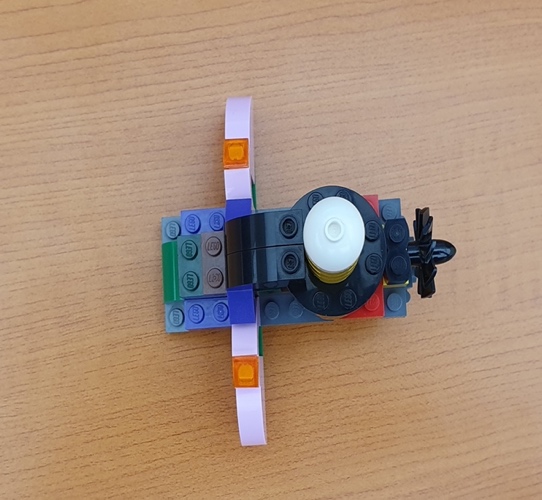 |  |

**Round 3, Week 6, Participant X2**

*Building challenge: Biodiversity*

|  | Grader 1 | Grader 2 | Grader 3 | Grader 4 | Grader 5 | Grader 6 | Grader 7 | AVE |
| --- | --- | --- | --- | --- | --- | --- | --- | --- |
|  | 1 | 1 | 1 | 1 | 2 | 0 | 1 | 1 |
| * note: Grader 6 subtracted 1 point for suspected plagiarism | | | | | | | | |

The rectangular bricks represent the number of plastics that is being used currently. The most direct way would be reducing the number of plastics used (represented by square bricks) by recycling plastics or using alternatives (paper containers) to replace plastics. In addition, degradable plastic material can help on the breakdown of plastics to smaller parts (smallest bricks in the model) which can prevent accumulation of plastics in the environment.

| 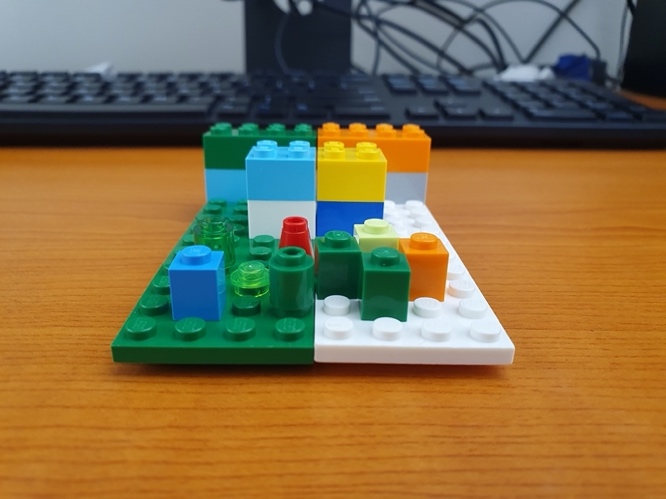 | 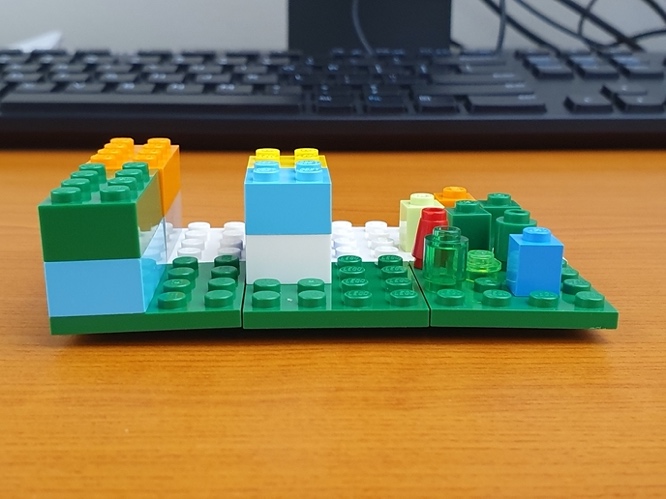 |
| --- | --- |
| 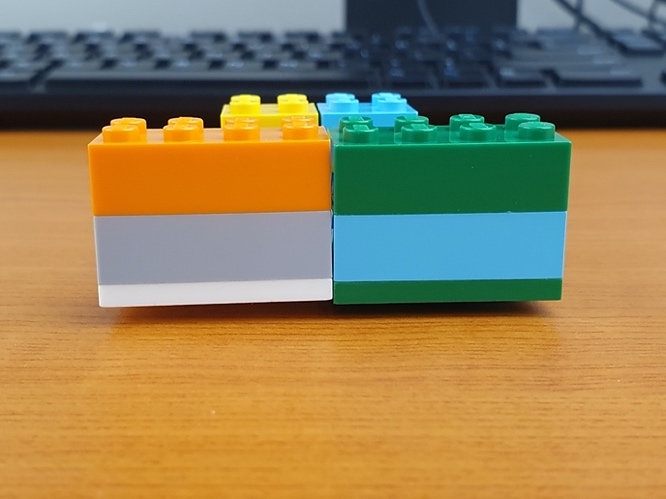 | 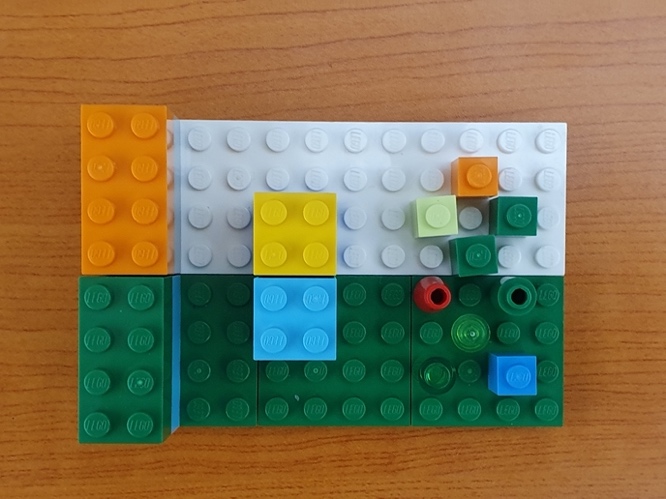 |
| 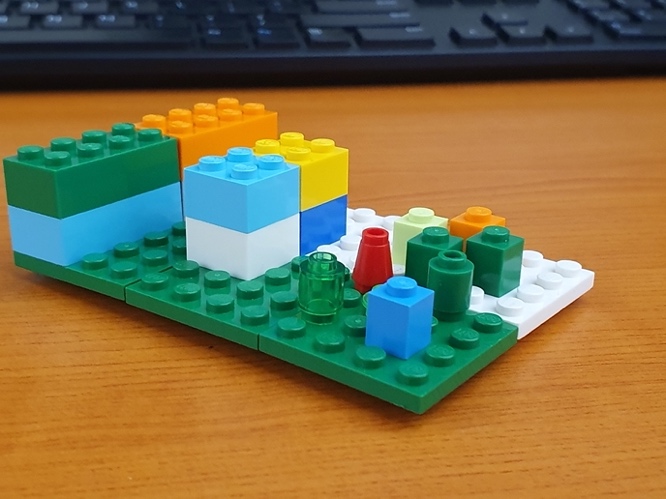 |  |
